# The endorsement of general and artificial intelligence reporting guidelines in radiological journals: a meta-research study

**DOI:** 10.1186/s12874-023-02117-x

**Published:** 2023-12-13

**Authors:** Jingyu Zhong, Yue Xing, Junjie Lu, Guangcheng Zhang, Shiqi Mao, Haoda Chen, Qian Yin, Qingqing Cen, Run Jiang, Yangfan Hu, Defang Ding, Xiang Ge, Huan Zhang, Weiwu Yao

**Affiliations:** 1 Department of Imaging, Tongren Hospital, Shanghai Jiao Tong University School of Medicine, Shanghai, 200336 China; 2 Department of Epidemiology and Population Health, Stanford University School of Medicine, Stanford, CA 94305 USA; 3 Department of Orthopedics, Shanghai Sixth People’s Hospital, Shanghai Jiao Tong University School of Medicine, Shanghai, 200233 China; 4 Department of Medical Oncology, Shanghai Pulmonary Hospital, Tongji University School of Medicine, Shanghai, 200433 China; 5 Department of General Surgery, Pancreatic Disease Center, Ruijin Hospital, Shanghai Jiao Tong University School of Medicine, Shanghai, 200025 China; 6 Department of Pathology, Shanghai Sixth People’s Hospital, Shanghai Jiao Tong University School of Medicine, Shanghai, 200233 China; 7 Department of Dermatology, Shanghai Ninth People’s Hospital, Shanghai Jiao Tong University School of Medicine, Shanghai, 200011 China; 8 Department of Pharmacovigilance, Shanghai Hansoh BioMedical Co., Ltd., Shanghai, 201203 China; 9 Department of Radiology, Ruijin Hospital, Shanghai Jiao Tong University School of Medicine, Shanghai, 200025 China

**Keywords:** Checklist, Guideline, Research report, Radiology, Artificial intelligence

## Abstract

**Background:**

Complete reporting is essential for clinical research. However, the endorsement of reporting guidelines in radiological journals is still unclear. Further, as a field extensively utilizing artificial intelligence (AI), the adoption of both general and AI reporting guidelines would be necessary for enhancing quality and transparency of radiological research. This study aims to investigate the endorsement of general reporting guidelines and those for AI applications in medical imaging in radiological journals, and explore associated journal characteristic variables.

**Methods:**

This meta-research study screened journals from the Radiology, Nuclear Medicine & Medical Imaging category, Science Citation Index Expanded of the 2022 Journal Citation Reports, and excluded journals not publishing original research, in non-English languages, and instructions for authors unavailable. The endorsement of fifteen general reporting guidelines and ten AI reporting guidelines was rated using a five-level tool: “active strong”, “active weak”, “passive moderate”, “passive weak”, and “none”. The association between endorsement and journal characteristic variables was evaluated by logistic regression analysis.

**Results:**

We included 117 journals. The top-five endorsed reporting guidelines were CONSORT (Consolidated Standards of Reporting Trials, 58.1%, 68/117), PRISMA (Preferred Reporting Items for Systematic Reviews and Meta-Analyses, 54.7%, 64/117), STROBE (STrengthening the Reporting of Observational Studies in Epidemiology, 51.3%, 60/117), STARD (Standards for Reporting of Diagnostic Accuracy, 50.4%, 59/117), and ARRIVE (Animal Research Reporting of In Vivo Experiments, 35.9%, 42/117). The most implemented AI reporting guideline was CLAIM (Checklist for Artificial Intelligence in Medical Imaging, 1.7%, 2/117), while other nine AI reporting guidelines were not mentioned. The Journal Impact Factor quartile and publisher were associated with endorsement of reporting guidelines in radiological journals.

**Conclusions:**

The general reporting guideline endorsement was suboptimal in radiological journals. The implementation of reporting guidelines for AI applications in medical imaging was extremely low. Their adoption should be strengthened to facilitate quality and transparency of radiological study reporting.

**Supplementary Information:**

The online version contains supplementary material available at 10.1186/s12874-023-02117-x.

## Introduction

Complete reporting is essential for translating results of clinical research into scientifically robust evidence to support decision-making in daily practice [1]. Reporting guidelines are developed to serve as useful tools to enhance the quality and transparency of clinical research [2, 3], while it is still a long-standing and widespread issue that the reporting quality is suboptimal [4, 5]. It is no wonder that a series of studies have repeatedly stressed the unsatisfied endorsement of reporting guideline in clinical journals [6–19], since the journals’ endorsement of reporting guidelines may raise the stakeholders’ awareness of them and is related to the completeness of reporting in medical journals [20].

The reporting quality of diagnostic accuracy tests and systematic reviews in *Radiology, European Radiology,* and *Korean Journal of Radiology* has been improved since it became mandatory to use the corresponding reporting guidelines [21–23]. It is reasonable that the change of the editorial policy of one specific journal significantly influences the reporting quality. On the other hand, the reporting quality of the abstracts of randomized controlled trials in the field of interventional radiology did not show obvious improvement after the update of corresponding guidelines [24]. One of the potential reasons for this difference is that the level of endorsement of reporting guidelines are different among the 61 interventional radiological journals that had been investigated. Therefore, it is necessary to investigate the endorsement of reporting guidelines, and sequent call those journals with low endorsement to make it mandatory. The earlier use of reporting guidelines during research process is considered to have greater impact on the final manuscript in radiological journals, suggesting that there is a need for enhanced education on the use of reporting guidelines [25]. The reporting guidelines should be introduced to the researchers to educate them about the benefits, be used by the reviewers during their review process, and be endorsed by journals to guarantee the adherence.

The artificial intelligence (AI) has engendered a rapid increasing medical application, especially in medical imaging [26–30]. The clinical translation of these academic research on AI should be based on scientific publication with enough details to allow readers to determine the rigor, quality, and generalizability. These publications of AI research in medical imaging calls for specific reporting guidelines for transparent and organized AI research reporting [31–35]. In addition to general reporting guidelines, the endorsement of these AI reporting guidelines has potential to improve the reporting quality and transparency of AI research, and reshape the current radiological practice. So far, the endorsement of AI reporting guidelines has not been assessed.

Therefore, this study aimed to investigate the endorsement of general reporting guidelines and those for AI applications in medical imaging in radiological journals, and explore associated journal characteristic variables.

## Materials and methods

### Study design

Our study is a meta-research study, and corresponding reporting guideline is currently not available [36–41]. Ethical approval or written informed consent were not required for this study because no human or animal subjects have been included in this study. We did not register the study protocol since there were no appropriate platform. However, we have drafted a protocol for this cross-sectional meta-research study (Supplementary Note S1). The sample selection, data extraction, and guideline endorsement assessment were conducted by two independent reviewers (JYZ and YX). The statistical analysis was performed by one reviewer (JYZ) under supervision of a methodologist (JJL). Any discrepancies will be resolved by discussion or consulting with the review group. Our study group is consisted of reviewers with diverse background and knowledge, including radiologists, and health professionals from multiple disciplines. All the reviewers have experience in manuscript drafting and publishing. Some of the reviewers have served as reviewers for radiological journals and evidence-based medicine journals, as well as editorial board members for radiological journals.

### Sample journals

The journals from the Radiology, Nuclear Medicine & Medical Imaging category, Science Citation Index Expanded of the 2022 Journal Citation Reports were identified via Clarivate website [42], and assessed for eligibility. The exclusion criteria were (1) journals not publishing original research; (2) journals published in non-English languages; and (3) journals lacking instructions for authors.

### Data extraction

The following bibliometrics information was directly downloaded via Clarivate: journal name, 2022 Journal Impact Factor (JIF), and the JIF quartile (Q1, Q2, Q3, Q4). The following journal characteristics were extracted: publication region, publication institution/publisher, publication language, publication frequency, type of access, whether the journal is only in the Radiology, Nuclear Medicine & Medical Imaging category, and whether the journal is owned by an academic society [43, 44]. The official website address of each journal was recorded. The search and data extraction via Clarivate was carried out on 20 July 2023.

### Endorsement assessment

The endorsement of fifteen general reporting guidelines [45–59] and ten reporting guidelines for AI applications in medical imaging [60–69] was rated using a 5-level tool [19]. The 15 general reporting guidelines were selected since they are considered as the most frequently used for main study types, and are highlighted on the Enhancing the QUAlity and Transparency Of health Research (EQUATOR) Network website [70]: (1) CONSORT (Consolidated Standards of Reporting Trials) for randomized trials [45], (2) STROBE (STrengthening the Reporting of Observational Studies in Epidemiology) for observational studies [46], (3) PRISMA (Preferred Reporting Items for Systematic Reviews and Meta-Analyses) for systematic reviews [47], (4) SPIRIT (Standard Protocol Items: Recommendations for Interventional Trials) for study protocols [48], (5) PRISMA-P (Preferred Reporting Items for Systematic Review and Meta-Analysis Protocols) for study protocols [49], (6) STARD (Standards for Reporting of Diagnostic Accuracy) for diagnostic/prognostic studies [50], (7) TRIPOD (Transparent Reporting of a multivariable prediction model for Individual Prognosis Or Diagnosis) for diagnostic/prognostic studies [51], (8) CARE (CAse REport guidelines) for case report [52], (9) AGREE (Appraisal of Guidelines, Research, and Evaluation) for clinical practice guidelines [53], (10) RIGHT (Reporting Items for Practice Guidelines in Healthcare) for clinical practice guidelines [54], (11) SRQR (Standards for Reporting of Qualitative Research) for qualitative research [55], (12) COREQ (COnsolidated criteria for REporting Qualitative research) for qualitative research [56], (13) ARRIVE (Animal Research Reporting of In Vivo Experiments) for animal pre-clinical studies [57], (14) SQUIRE (Standards for QUality Improvement Reporting Excellence) for quality improvement studies [58], and (15) CHEERS (Consolidated Health Economic Evaluation Reporting Standards) for economic evaluations [59]. The 10 reporting guidelines for AI applications in medical imaging were identified and chosen according to relevant reviews and expert’s opinions [31–35]: (1) CONSORT-AI (Consolidated Standards of Reporting Trials involving Artificial Intelligence) [60], (2) SPIRIT-AI (Standard Protocol Items: Recommendations for Interventional Trials involving Artificial Intelligence) [61], (3) FUTURE-AI (Fairness Universality Traceability Usability Robustness Explainability Artificial Intelligence solutions) [62], (4) MI-CLAIM (Minimum Information about Clinical Artificial Intelligence Modeling) [63], (5) MINIMAR (Minimum Information for Medical AI Reporting) [64], (6) CLAIM (CheckList for Artificial Intelligence in Medical imaging) [65], (7) MAIC-10 (Must Artificial Intelligence Criteria-10) [66], (8) RQS (Radiomics Quality Score) [67], (9) IBSI (Image Biomarker Standardization Initiative) [68], (10) CLEAR (CheckList for EvaluAtion of Radiomics research) [69]. The reporting guidelines included in our study did not cover all the available AI reporting guidelines. Therefore, we also recorded the extra identified AI reporting guidelines if the journal websites mentioned it. Unfortunately, there was no extra AI reporting guidelines identified.

The modified 5-level tool rated the endorsement into: (1) “active strong”: journal requires completed checklist and/or flow diagram with article submission; (2) “active weak”: journal encourages to flow a specific guideline; (3) “passive moderate”: journal only requires the abstract to follow a specific guideline; (4) “passive weak”: journal encourages to prepare manuscripts according to EQUATOR Network website [70] or the International Committee of Medical Journal Editors document [71]; and (5) “none”: journal does not mention any reporting guideline [19]. The assessment on journal’s endorsement of reporting guidelines were based on their documents on submission (instructions for authors, submission guideline, editorial policies, etc.). The endorsement of reporting guidelines was assessed from 22 July 2023 to 23 July 2023.

### Statistical analysis

The statistical analysis was conducted by using R language version 4.1.3 within RStudio version 1.4.1106 [72–75]. The endorsement types of “active strong”, “active weak”, “passive moderate”, and “passive weak” were considered as a positive outcome, while the type of “none” was treated as a negative outcome. The journal was considered as positive if at least one reporting guideline was positive; otherwise, the journal was treated as negative. The journal characteristics between the positive and negative groups were compared. The journal characteristics were further evaluated by logistic regression analysis to tell whether they are associated with the reporting guidelines endorsement. The factors associated with the endorsement of general reporting guidelines and those for AI applications in medical imaging were assessed together, because the endorsement of reporting guidelines for AI applications in medical imaging were very low. All of the statistical tests were two-sided. The alpha level for statistically significance is set at 0.05, if not stated otherwise.

## Results

### Journal inclusion

**Fig. 1:**
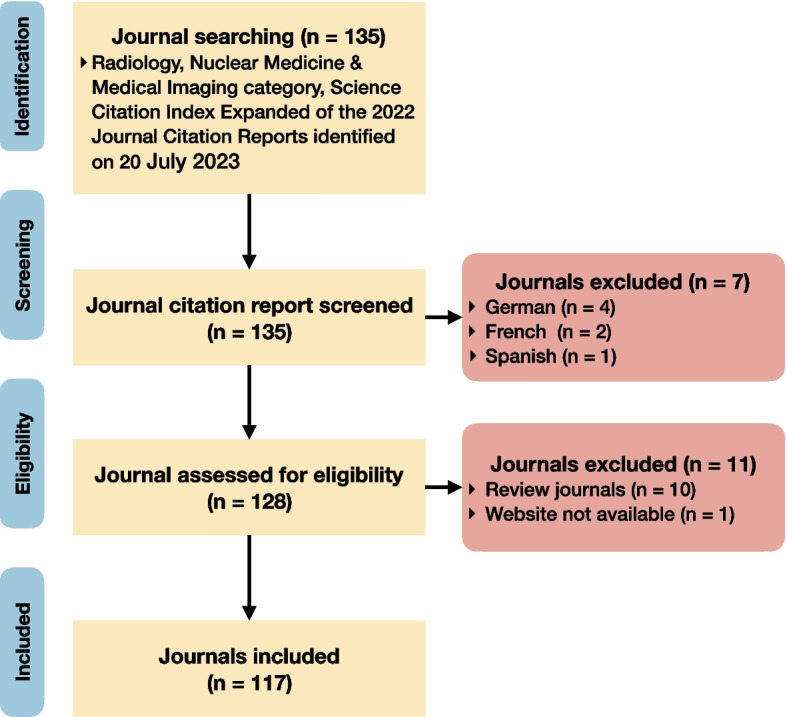
Flowchart for radiological journal inclusion

There were 135 journals in the Radiology, Nuclear Medicine & Medical Imaging category, Science Citation Index Expanded of the 2022 Journal Citation Reports. We excluded 10 journals that only publish reviews, 7 non-English journals, and 1 journal without available website. Finally, 117 radiological journals were included (Fig. 1).

### Journal characteristics

**Fig. 2:**
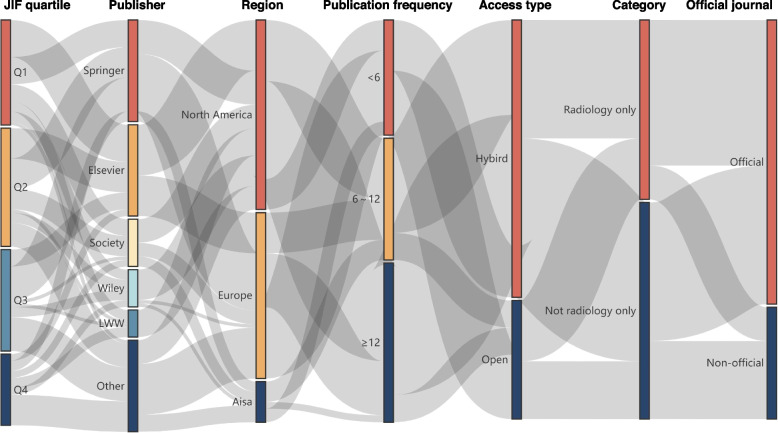
Sankey diagram of journal characteristics. Abbreviation: JIF = Journal Impact Factor, LWW = Lippincott Williams & Wilkins

The mean ± standard deviation, median (range) of 2022 JIF of included journals was 3.7 ± 2.8, 2.9 (0.6 to 19.7) (Table 1). The journals were most likely belonged to JIF Q2 (29.9%, 35/117), North America (47.9%, 56/117), and not only included in the Radiology, Nuclear Medicine & Medical Imaging category (54.7%, 64/117). Most of the journals were published by Springer (25.6%, 30/117), with a frequency of ≥ 12 issue/year (40.2%, 47/117), supporting hybrid access mode (70.1%, 82/117), and were owned by academic societies (71.8%, 84/117) (Fig. 2). The characteristics of each journal are presented in Supplementary Tables S1 and S2. The original data for analysis are presented in Supplementary Data Sheet.

**Table 1:** Characteristics of included radiological journals

Characteristics	All (*N* = 117)	Positive (*N* = 72)	Negative (*N* = 45)	*P* value
2022 JIF, mean ± SD, median (range)	3.7 ± 2.8, 2.9 (0.6 to 19.7)	3.8 ± 2.7, 3.1 (0.6 to 19.7)	3.5 ± 2.9, 2.5 (0.6 to 14.0)	0.552
JIF quartile, n (%)				
Q1	31 (26.5)	22 (30.6)	9 (20.0)	0.208
Q2	35 (29.9)	23 (31.9)	12 (26.7)	
Q3	30 (25.6)	18 (25.0)	12 (26.7)	
Q4	21 (17.9)	9 (12.5)	12 (26.7)	
Publisher, n (%)				
Springer	30 (25.6)	27 (37.5)	3 (6.7)	< 0.001
Elsevier	27 (23.1)	13 (18.1)	14 (31.1)	
Society	14 (12.0)	8 (11.1)	6 (13.3)	
Wiley	11 (9.4)	4 (5.6)	7 (15.6)	
LWW	8 (6.8)	1 (1.4)	7 (15.6)	
Other	27 (23.1)	19 (26.4)	8 (17.8)	
Region, n (%)				
North America	56 (47.9)	30 (41.7)	26 (57.8)	0.077
Europe	49 (41.9)	36 (50.0)	13 (28.9)	
Asia	12 (10.3)	6 (8.3)	6 (13.3)	
Publication frequency, n (%)				
≥ 12 issue/year	47 (40.2)	29 (40.3)	18 (40.0)	0.860
6–12 issue/year	36 (30.8)	21 (29.2)	15 (33.3)	
< 6 issue/year	34 (29.1)	22 (30.6)	12 (26.7)	
Type of access, n (%)				
Hybrid	82 (70.1)	50 (69.4)	32 (71.1)	0.848
Open	35 (29.9)	22 (30.6)	13 (28.9)	
Only in Radiology category, n (%)				
Yes	53 (45.3)	35 (48.6)	18 (40.0)	0.363
No	64 (54.7)	37 (51.4)	27 (60.0)	
Official journal, n (%)				
Yes	84 (71.8)	55 (76.4)	29 (64.4)	0.162
No	33 (28.2)	17 (23.6)	16 (35.6)	

The endorsement types of “active strong”, “active weak”, “passive moderate”, and “passive weak” were considered as a positive outcome, while the type of “none” was treated as a negative outcome. The independent t test was used for comparing the 2022 JIF between positive and negative groups, while the chi-square tests were used for comparing other characteristics between positive and negative groups

*Abbreviation*: *SD* standard deviation, *JIF* journal impact factor, *LWW* Lippincott Williams & Wilkins

### Endorsement of reporting guidelines

The journals were divided into positive (61.5%, 72/117) and negative (38.5%, 45/117) groups (Table 1). The endorsement level of general reporting guidelines was most likely to be “active weak” (12.8% to 32.5%, 15/117 to 38/117) (Table 2). The top-five general reporting guidelines were CONSORT (58.1%, 68/117), PRISMA (54.7%, 64/117), STROBE (51.3%, 60/117), STARD (50.4%, 59/117), and ARRIVE (35.9%, 42/117).

**Table 2:** Endorsement levels of reporting guidelines in radiological journals

Endorsement level	Active strong, n (%)	Active weak, n (%)	Passive moderate, n (%)	Passive weak, n (%)	None, n (%)
General reporting guidelines					
CONSORT	27 (23.1)	32 (27.4)	2 (1.7)	8 (6.8)	49 (41.9)
STROBE	7 (6.0)	34 (29.1)	2 (1.7)	17 (14.5)	57 (48.7)
PRISMA	14 (12.0)	38 (32.5)	2 (1.7)	10 (8.5)	53 (45.3)
SPIRIT	0 (0.0)	22 (18.8)	0 (0.0)	16 (13.7)	79 (67.5)
PRISMA-P	0 (0.0)	17 (14.5)	2 (1.7)	19 (16.2)	79 (67.5)
STARD	9 (7.7)	33 (28.2)	2 (1.7)	15 (12.8)	58 (49.6)
TRIPOD	2 (1.7)	23 (19.7)	0 (0.0)	15 (12.8)	77 (65.8)
CARE	3 (2.6)	22 (18.8)	0 (0.0)	15 (12.8)	77 (65.8)
ARRIVE	4 (3.4)	27 (23.1)	0 (0.0)	11 (9.4)	75 (64.1)
AGREE	0 (0.0)	17 (14.5)	0 (0.0)	21 (17.9)	79 (67.5)
RIGHT	1 (0.9)	15 (12.8)	0 (0.0)	22 (18.8)	79 (67.5)
SRQR	2 (1.7)	18 (15.4)	0 (0.0)	20 (17.1)	78 (66.7)
COREQ	0 (0.0)	21 (17.9)	0 (0.0)	17 (14.5)	79 (67.5)
SQUIRE	0 (0.0)	20 (17.1)	0 (0.0)	20 (17.1)	77 (65.8)
CHEERS	1 (0.9)	24 (20.5)	0 (0.0)	14 (12.0)	78 (66.7)
Reporting guidelines for AI applications in medical imaging					
CONSRT-AI	0 (0.0)	0 (0.0)	0 (0.0)	0 (0.0)	0 (0.0)
SPIRIT-AI	0 (0.0)	0 (0.0)	0 (0.0)	0 (0.0)	0 (0.0)
FUTURE-AI	0 (0.0)	0 (0.0)	0 (0.0)	0 (0.0)	0 (0.0)
MI-CLAIM	0 (0.0)	0 (0.0)	0 (0.0)	0 (0.0)	0 (0.0)
MINIMAR	0 (0.0)	0 (0.0)	0 (0.0)	0 (0.0)	0 (0.0)
CLAIM	0 (0.0)	2 (1.7)	0 (0.0)	0 (0.0)	0 (0.0)
MAIC-10	0 (0.0)	0 (0.0)	0 (0.0)	0 (0.0)	0 (0.0)
RQS	0 (0.0)	0 (0.0)	0 (0.0)	0 (0.0)	0 (0.0)
IBSI	0 (0.0)	0 (0.0)	0 (0.0)	0 (0.0)	0 (0.0)
CLEAR	0 (0.0)	0 (0.0)	0 (0.0)	0 (0.0)	0 (0.0)

**Fig. 3:**
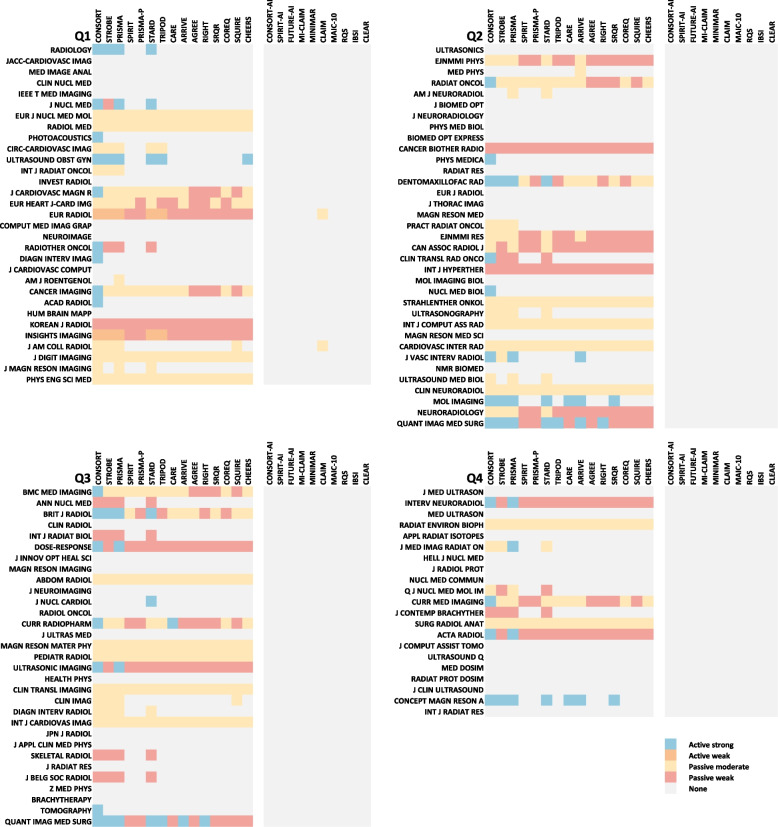
Endorsement of each reporting guideline according to radiological journals. The left part presents the endorsement of 15 general reporting guidelines. The right part presents the endorsement of 10 reporting guidelines for AI applications in medical imaging. Abbreviation: Q1 to Q4 = the first to the forth Journal Impact Factor quartile

The most implemented reporting guidelines for AI applications in medical imaging was CLAIM (1.7%, 2/117), while other nine artificial intelligence reporting guidelines were not mentioned in documents on submission of radiological journals. The examples for five levels of endorsement are presented in Supplementary Table S3. The endorsement of reporting guidelines of each journal is presented in Fig. 3 and Supplementary Table S4.

### Factors associated with the endorsement of reporting guidelines

The journal characteristics did not show difference between the positive and negative groups, except for the distribution of publisher (Table 1). The multivariable logistic regression analysis showed that JIF quartile and publisher were associated with the endorsement of reporting guidelines in radiological journals (Table 3). The JIF Q2 journals were more likely to endorse the reporting guidelines than JIF Q1 journals (odds ratio 7.83, 95% confidence interval 1.70–36.20, *P* = 0.008). The journals that are published by academic societies (0.17, 0.04–0.64, *P* = 0.009), Lippincott Williams & Wilkins (0.13, 0.03–0.68, *P* = 0.016), and other publishers (0.03, 0.002–0.33, *P* = 0.004) were less likely to endorse the reporting guidelines than those published by Springer.

**Table 3:** Factors associated with the endorsement of reporting guidelines

Variable grouping	Univariable logistic analysis			Multivariable logistic analysis		
	**OR**	**95% CI**	***P* value**	**OR**	**95% CI**	***P* value**
JIF quartile						
Q1	1.00	1.00–1.00		1.00	1.00–1.00	
Q2	3.26	1.02–10.41	0.046	7.83	1.70–36.20	0.008
Q3	2.56	0.84–7.76	0.098	3.53	0.88–14.21	0.076
Q4	2.00	0.65–6.20	0.230	1.60	0.42–6.17	0.495
Publisher						
Springer	1.00	1.00–1.00		1.00	1.00–1.00	
Elsevier	3.79	0.89–16.17	0.072	2.59	0.57–11.65	0.216
Society	0.39	0.13–1.20	0.100	0.17	0.04–0.64	0.009
Wiley	0.56	0.15–2.15	0.399	0.26	0.06–1.20	0.084
LWW	0.24	0.06–1.06	0.059	0.13	0.03–0.68	0.016
Other	0.06	0.01–0.57	0.014	0.03	0.002–0.33	0.004
Region						
North America	1.00	1.00–1.00		n. a		
Europe	1.15	0.33–4.02	0.822	n. a		
Asia	2.77	0.78–10.13	0.124	n. a		
Publication frequency						
≥ 12 issue/year	1.00	1.00–1.00		n. a		
6–12 issue/year	0.88	0.35–2.20	0.782	n. a		
< 6 issue/year	0.76	0.29–2.01	0.584	n. a		
Type of access						
Hybrid	1.00	1.00–1.00		n. a		
Open	0.92	0.41–2.09	0.848	n. a		
Only in Radiology category						
Yes	1.00	1.00–1.00		n. a		
No	1.42	0.67–3.02	0.363	n. a		
Official journal						
Yes	1.00	1.00–1.00		n. a		
No	1.79	0.79–4.04	0.165	n. a		

*Abbreviation*: *CI* confidence interval, *LWW* Lippincott Williams & Wilkins, *OR* odds ratio

## Discussion

The endorsement of general reporting guidelines was lowest for SPRIT, PRISMA-P, AGREE, RIGHT, and COREQ (all 32.5%) in radiological journals, and highest for CONSORT (58.1%). Only two journals suggested to implant the CLAIM (1.7%), while the other nine evaluated reporting guidelines for AI applications in medical imaging were not mentioned. The JIF quartile and publisher were associated with the endorsement of reporting guidelines in radiological journals.

The CONSORT statement was one of the most early developed reporting guidelines for randomized clinical trials, followed by a brunches of reporting guidelines published covering main study types. In accordance to the previous studies, the CONSORT statement has been most commonly endorsed by journals in varying specialties [6–19]. The methodology of randomized clinical trials was less difference among journals from different specialties, and the CONSORT statement met the requirements for most of the journals. Likewise, the reporting guidelines for systematic reviews, observational studies, and animal studies were also with relatively high endorsement, because their methodology were also of less variety. The radiological journals further recommend to use STARD statement, since the diagnostic accuracy test plays an important role in radiology [21, 22]. However, the TRIPOD statement has not been widely accepted by the radiological journals. It is possible that the study type of multivariable prediction model is less conducted than the diagnostic accuracy test. The reporting guidelines for other study types were less endorsed by radiological journals. It is possible that the protocol, and case report are not an acceptable study type for some of the journals, and guidelines, qualitative research, qualitative research, quality improvement, and economic evaluations were not common study types for radiological journals.

In addition to general reporting guidelines, the reporting guidelines for AI applications in medical imaging were also evaluated in our study. Unfortunately, the endorsement of investigated AI reporting guidelines was extremely low in radiological journal. Only *European Radiology* and *Journal of the American College of Radiology* recommended CLAIM for AI studies, although the CLAIM was published on the *Radiology: Artificial Intelligence* [65]. The MAIC-10 and CLEAR were two guidelines recently published on *Insights into Imaging* for AI studies and radiomics studies [66, 69], and the IBSI statement for radiomics was introduced by *Radiology* [68]. However, they have not been widely implanted by radiological journals even the journals published them. In contrast to the low endorsement in radiological journals, these reporting guidelines were usually applied for systematic reviews [76–85]. These systematic reviews found that the adherence rate of RQS, IBSI, and CLAIM were suboptimal for radiomics and AI studies for medical imaging. It is not weird since most of the radiological journals did not endorse these reporting guidelines. The reporting transparency has been improved after the introduction of reporting guidelines [20–23]. It is expectable to make the reporting guidelines for AI applications in medical imaging mandatory to improve the awareness and application of them, in order to achieve transparent AI and radiomics study reporting. The AI and radiomics community should understand the importance of proper self-reporting, and encourage researchers, journals, editors, and reviewers to take action to ensure the proper usage of checklists [82–85]. We plan to investigate the influence of the endorsement of the reporting guidelines for AI applications in medical imaging on the quality of study reporting.

We found that the JIF quartile and publisher were associated with the endorsement of reporting guidelines in radiological journals. The journals with higher JIF were generally more likely to endorse the reporting guidelines than lower ones [6, 15, 17, 18]. It is reasonable that the journals with higher impact factors have higher endorsement of reporting guidelines, since they were considered to have higher quality. However, our study showed that the JIF Q2 journals were more likely to endorse the reporting guidelines than JIF Q1 ones in radiological journals. The underlying reason is not yet clear. The journals that were published by Springer showed higher endorsement of reporting guidelines in both surgery and radiological journals [17]. We infer that the higher endorsement was benefited by the unified editorial policy of Springer that recommended the specific reporting guidelines and EQUATOR Network website. The surgical journals from United Kingdom and Europe were more likely to endorse the reporting guidelines than those from North America [17], but we did not find the influence of publication region in radiological journals.

The requirement of adherence to reporting guidelines may improve reporting quality [20–23], and the agreement among journals on the endorsement of reporting guidelines could improve the quality of research publishing [86]. Rather than prioritizing additional studies on the poor quality of health research reporting, interventions are needed to improve reporting [87]. However, only a limited number of tools has been raised for this purpose, and a smaller number of them has been evaluated [88]. There was the only one that showed a statistically significant effect on reporting quality was a Consort-based WEB tool [89, 89]. This tool supports adherence at the manuscript writing stage. The earlier the reporting guidelines were used, the more impact on the final manuscript and higher perceived value [25]. Therefore, it has been repeatedly suggested to enhanced education on the use of these guidelines. It seemed to be most pivotal to support journals to include hard-wiring adherence to reporting guidelines into their editorial policy. It may be an effective way to make the reporting guidelines mandatory, and ask the reviewers to use the reporting guidelines during the review process. The reporting quality has been improved if the journal required authors to incorporate section headings that reflected CONSORT items into their manuscripts [90]. Although it has not been found that these interventions are effective in radiological journals, the authors, reviewers, and editors may actively use the reporting guideline to guide themselves in the study design, conduction, drafting, reviewing, and revision. Further survey is need to identify the obstacles against the endorsement of reporting guidelines in journals. The potential reasons for suboptimal endorsement of reporting guidelines includes the insufficient resources for mandatory use, the needs to reduce barriers to submission and review, the unique editorial perspective, or the use of alternative to improve the study quality [16, 17]. As the current study may aware the radiological community on the issue of suboptimal endorsement of reporting guidelines. We plan to re-evaluate the endorsement in the future to find out whether the publication of radiological research is reshaped.

Our study has limitations. First, our study only included the radiological journals in the Science Citation Index Expanded. We selected these radiological journals as a representative sample of the high-quality journals of this field. Our results may over-estimate the journals’ endorsement of the reporting guideline. Second, our study is a cross-sectional study only presenting the current endorsement of reporting guidelines in radiological journals is suboptimal. An update study should be conducted in the future. Third, we relied on online documents to assess the endorsement without verifying the additional instructions potentially appear during the manuscript submission and review process. Although we collected and cross-checked the journal information, the journal websites may be update afterwards. Changes in editorial policies may not be timely updated online, which may influence on our conclusion. Fourth, we only investigated a limited number of general reporting guidelines and those for AI applications in medical imaging. There are a lot of reporting guidelines developed for more specific purpose and are available on EQUATOR Network website. Although our study did not cover all the available guidelines, we considered that the included reporting guidelines can at least represent the usually-used ones. We did find many extra AI guidelines which was not included in our study [91–95]. It would be interesting to investigate the endorsement of AI reporting guidelines which is not specifically designed for AI application medical imaging. Finally, our study only emphasized the potential of making reporting guidelines for AI applications in medical imaging mandatory to improve awareness and application in order to achieve high-quality study reporting. Nevertheless, there are currently too many different reporting guidelines for the AI research domain that is difficult for authors, reviewers, and editors to choose [31–35, 60–69, 91–95]. It is the next step of study to assess how relevant and how actual are these developed and developing guidelines to the current state of development of AI.

## Conclusion

As a summary, our study found that the general reporting guideline endorsement are suboptimal in radiological journals. The implementation of artificial intelligence reporting guidelines was extremely low. Radiological journals may consider making general and artificial intelligence reporting guidelines mandatory to improve their awareness and application, in order to achieve high-quality and transparent radiological study reporting.

### Supplementary Information

**Additional file 1:** **Supplementary Note S1.** Study protocol. **Supplementary Table S1.** Bibliometrics information of included and excluded journals. **Supplementary Table S2.** Characteristics and homepages of included and excluded journals. **Supplementary Table S3.** Five endorsement level defined with examples in radiological journals. **Supplementary Table S4.** Endorsement level rating of reporting guidelines of included journals.

**Additional file 2. **Data Sheet.

## Data Availability

All data generated or analysed during this study are included in this published article and its supplementary information files.

## Abbreviations

AGREE: Appraisal of Guidelines, Research, and Evaluation
AI: Artificial Intelligence
ARRIVE: Animal Research Reporting of In Vivo Experiments
CARE: CAse REport guidelines
CHEERS: Consolidated Health Economic Evaluation Reporting Standards
CLAIM: CheckList for Artificial Intelligence in Medical imaging
CLEAR: CheckList for EvaluAtion of Radiomics research
CONSORT: Consolidated Standards of Reporting Trials
CONSORT-AI: Consolidated Standards of Reporting Trials involving Artificial Intelligence
COREQ: COnsolidated criteria for REporting Qualitative research
EQUATOR: Enhancing the QUAlity and Transparency Of health Research
FUTURE-AI: Fairness Universality Traceability Usability Robustness Explainability Artificial Intelligence solutions
IBSI: Image Biomarker Standardization Initiative
JIF: Journal Impact Factor
MAIC-10: Must Artificial Intelligence Criteria-10
MI-CLAIM: Minimum Information about Clinical Artificial Intelligence Modeling
MINIMAR: Minimum Information for Medical AI Reporting
PRISMA: Preferred Reporting Items for Systematic Reviews and Meta-Analyses
PRISMA-P: Preferred Reporting Items for Systematic Review and Meta-Analysis Protocols
RIGHT: Reporting Items for Practice Guidelines in Healthcare
RQS: Radiomics Quality Score
SPIRIT: Standard Protocol Items: Recommendations for Interventional Trials
SPIRIT-AI: Standard Protocol Items: Recommendations for Interventional Trials involving Artificial Intelligence
SQUIRE: Standards for QUality Improvement Reporting Excellence
SRQR: Standards for Reporting of Qualitative Research
STARD: Standards for Reporting of Diagnostic Accuracy
STROBE: STrengthening the Reporting of Observational Studies in Epidemiology
TRIPOD: Transparent Reporting of a multivariable prediction model for Individual Prognosis Or Diagnosis
