# Supplementary material for: The endorsement of general and artificial intelligence reporting guidelines in radiological journals: a meta-research study
Source: BMC Med Res Methodol. 2023 Dec 13;23:292. doi: 10.1186/s12874-023-02117-x (PMC10717715; doi:10.1186/s12874-023-02117-x)
Supplement: Supplementary file 1 — Additional file 1: Supplementary Note S1. Study protocol. Supplementary Table S1. Bibliometrics information of included and excluded journals. Supplementary Table S2. Characteristics and homepages of included and excluded journals. Supplementary Table S3. Five endorsement level defined with examples in radiological journals. Supplementary Table S4. Endorsement level rating of reporting guidelines of included journals. [file 12874_2023_2117_MOESM1_ESM.docx]

**Supplementary Material**

**Title:** The endorsement of general and artificial intelligence reporting guidelines in radiological journals: a meta-research study

**List of Supplementary Material**

Supplementary Note S1 Study protocol

Supplementary Table S1 Bibliometrics information of included and excluded journals

Supplementary Table S2 Characteristics and homepages of included and excluded journals

Supplementary Table S3 Five endorsement level defined with examples in radiological journals

Supplementary Table S4 Endorsement level rating of reporting guidelines of included journals

**Supplementary Note S1 Study protocol**

**First drafted date:** 10 June 2023

**Last modification date:** 25 July 2023

**Study rationale**

Quality and transparency are essential for radiological study, which can promote the results of academic studies as clinical evidence, thus affecting the decision-making of clinical practice. Reporting guidelines have been proven to be useful tools for enhancing the quality and transparency of medical and radiological study [1-5]. Reporting guidelines have more author-perceived impact on the final manuscript and higher perceived value the earlier they were used, suggesting that there is a need for enhanced education on the use of these guidelines [6]. There is a brunch of reporting guidelines published for main study types [7-21]. However, the endorsement of reporting guidelines in different field of journals were suboptimal [22-35]. Further, their endorsement in radiological journals is unclear. On the other hand, there are many reporting guidelines developed for artificial intelligence study [36-45]. However, their endorsement has not been evaluated.

**Objectives**

Our study is aimed to investigate the endorsement of reporting guidelines in radiological journals and explore associated journal characteristic variables.

**Study design**

We did not register the study protocol since there were no appropriate platform. However, we drafted this protocol for this cross-sectional study, and will conduct it according to relevant guidelines, including STrengthening the Reporting of Observational Studies in Epidemiology (STROBE) [8].

**Study group**

Our study group is consisted of reviewers with diverse background and knowledge, including radiologists (ZJY, YX, YFH, DFD, XG, HZ, WWY), and health professionals from multiple disciplines (JJL, methodologist in epidemiology and population health; GCZ, orthopedist; SQM, oncologist; HDC, surgeon; QY, pathologist; QQC, dermatologist; RJ, expert in pharmacovigilance). All the reviewers have experience in manuscript drafting and publishing. Some of the reviewers (JYZ, YX, YFH, DFD, WWY, and HZ) have served as reviewers for radiological journals. Dr. Jingyu Zhong served as a reviewer for an evidence-based medicine journal (*Chinese Journal of Evidence-Based Medicine*, <http://www.cjebm.com/journal/zgxzyxzz>), as well as serve as an editorial board member for radiological journals (*European Radiology,* <https://www.springer.com/journal/330/>; *BMC Medical Imaging*, <https://bmcmedimaging.biomedcentral.com>).

**Sample journals**

The journals from the Radiology, Nuclear Medicine & Medical Imaging category, Science Citation Index Expanded of the 2022 Journal Citation Reports were identified via Clarivate (<https://jcr.clarivate.com/jcr/home>) by a reviewer (JYZ) with experience in literature searching. The journals will be screened and assessed for eligibility by two independent reviewers (JYZ and YX). Any discrepancies will be resolved by discussion or consulting with the review group.

The journals will be screened for eligibility according to the exclusion criteria:

(1) journals not publishing original research. The journals not publishing original research will be identified by the journal description, and further confirmed by screening the recent publications.

(2) journals in non-English languages. The publication language of journal will be defined according to the Journal Citation Reports. If the publication language of journal is multi-language, we will further confirm the publication language of this journal to decide whether the journal should be included or excluded. If the publication language of journal is non-English (e. g., German, French), the journal will be excluded. If the publication language of journal is English, the journal will be included.

(3) journals lacking instructions for authors. The journal will be excluded if the official website is unavailable, or the instruction for authors, author guidelines, peer reviewer guidance, editorial policies, or other relevant directions for authors of a journal is unavailable.

**Data extraction**

The bibliometrics information of included journals will be downloaded directly from the Radiology, Nuclear Medicine & Medical Imaging category, Science Citation Index Expanded of the 2022 Journal Citation Reports were identified via Clarivate (<https://jcr.clarivate.com/jcr/home>) by a reviewer (JYZ) with experience in literature searching. The homepages of included journals will be identified by the same reviewer (JYZ). The data extraction will be conducted and cross-checked by two independent reviewers (JYZ and YX). Any discrepancies will be resolved by discussion or consulting with the review group.

The following data will be extracted:

(1) Bibliometrics information: journal name, JCR abbreviation, ISSN, eISSN, 2022 journal impact factor (JIF) from Science Citation Index (SCI)/Science Citation Index Expanded (SCIE), the JIF quartile (Q1, Q2, Q3, Q4), JIF rank, total citations, and citable items.

(2) Journal characteristics: publication region (North America, European, Asia), publication institution/publisher (Elsevier, Lippincott Williams & Wilkins, Springer, Wiley, Oxford, etc.), publication language (only English available), publication frequency (monthly, bi-monthly, quarterly, etc.), type of access (conventional, open, or hybrid), whether the journal is only in the Radiology, Nuclear Medicine & Medical Imaging category (yes, no), whether the journal is owned by a professional society or institute (yes, no), and official website address of each journal.

**Endorsement assessment**

Our study will assess the endorsement level of fifteen general reporting guidelines [7-21] and ten artificial intelligence reporting guidelines [36-45] by using a 5-level tool [35]. The endorsement level rating of reporting guidelines of included journals will be conducted by two independent reviewers (JYZ and YX). Any discrepancies will be resolved by discussion or consulting with the review group.

We included fifteen general reporting guidelines [7-21]. These reporting guidelines are selected because they are highlighted by the Equator Network website (<https://www.equator-network.org>) as the reporting guidelines of main study types. The fifteen general reporting guidelines are:

(1) CONSORT (Consolidated Standards of Reporting Trials) for randomised trials.

(2) STROBE (STrengthening the Reporting of Observational Studies in Epidemiology) for observational studies.

(3) PRISMA (Preferred Reporting Items for Systematic Reviews and Meta-Analyses) for systematic reviews.

(4) SPIRIT (Standard Protocol Items: Recommendations for Interventional Trials) for study protocols.

(5) PRISMA-P (Preferred Reporting Items for Systematic Review and Meta-Analysis Protocols) for study protocols.

(6) STARD (Standards for Reporting of Diagnostic Accuracy) for diagnostic/prognostic studies.

(7) TRIPOD (Transparent Reporting of a multivariable prediction model for Individual Prognosis Or Diagnosis) for diagnostic/prognostic studies.

(8) CARE (CAse REport guidelines) for case report.

(9) AGREE (Appraisal of Guidelines, Research, and Evaluation) for clinical practice guidelines.

(10) RIGHT (Reporting Items for Practice Guidelines in Healthcare) for clinical practice guidelines.

(11) SRQR (Standards for Reporting of Qualitative Research) for qualitative research.

(12) COREQ (COnsolidated criteria for REporting Qualitative research) for qualitative research.

(13) ARRIVE (Animal Research Reporting of In Vivo Experiments) for animal pre-clinical studies.

(14) SQUIRE (Standards for QUality Improvement Reporting Excellence) for quality improvement studies.

(15) CHEERS (Consolidated Health Economic Evaluation Reporting Standards) for economic evaluations.

We included ten artificial intelligence reporting guidelines [36-45]. These reporting guidelines are selected because they are highlighted by reviews concerning on the artificial intelligence reporting guidelines in imaging and medical study [46-49]. A recently developed MAIC-10 (Must AI Criteria-10), IBSI (Image Biomarker Standardization Initiative), and CLEAR (CheckList for EvaluAtion of Radiomics research) are also included after consulting with experts [42,44,45] (HZ and WWY). The ten artificial intelligence reporting guidelines are:

(1) CONSORT-AI (Consolidated Standards of Reporting Trials involving Artificial Intelligence)

(2) SPIRIT-AI (Standard Protocol Items: Recommendations for Interventional Trials involving Artificial Intelligence)

(3) FUTURE-AI (Fairness Universality Traceability Usability Robustness Explainability Artificial Intelligence solutions)

(4) MI-CLAIM (Minimum Information about Clinical Artificial Intelligence Modeling)

(5) MINIMAR (Minimum Information for Medical AI Reporting)

(6) CLAIM (CheckList for Artificial Intelligence in Medical imaging)

(7) MAIC-10 (Must Artificial Intelligence Criteria-10)

(8) RQS (Radiomics Quality Score)

(9) IBSI (Image Biomarker Standardization Initiative)

(10) CLEAR (CheckList for EvaluAtion of Radiomics research)

The 5-level tool rates the endorsement level of reporting guidelines into [35]:

(1) Active strong: A requirement of a completed checklist and/or a flow diagram with article submission (e.g., “must”, “should be uploaded”).

(2) Active weak: A suggestion that authors are “encouraged” or “should” reference or follow a specific guideline; Priority publication if follow a specific guideline.

(3) Passive moderate: A suggestion that authors should adhere to “relevant” reporting guidelines; Abstracts are required to follow a specific guideline.

(4) Passive weak: References documents (e.g., ICMJE, or EQUATOR Network) which mention reporting guidelines. If the instructions for authors mentions the ICMJE (<https://www.icmje.org>), the 4 general reporting guidelines mentioned by ICMJE (CONSORT, STROBE, PRISMA, and STARD) will be rated as “Passive weak”. However, the mention of ICMJE in a specific section (such as authorship, conflict of interest, ethics, etc.), were not considered as appropriate. If the instructions for authors mentions the EQUATOR Network (<https://www.equator-network.org>), the 15 general reporting guidelines for main study types on the homepage of EQUATOR Network will be rated as “Passive weak”.

(5) None: No mention of any reporting guidelines.

The endorsement levels of reporting guidelines including “active strong”, “active weak”, “passive moderate”, and “passive weak” were used as a positive outcome. The endorsement level of reporting guidelines, “none” was considered as a negative outcome. The endorsement for all 23 reporting guidelines, endorsement for 15 general reporting guidelines, and endorsement for 8 artificial intelligence reporting guidelines, will be calculated respectively.

**Statistical analysis**

The statistical analysis will be performed with R language version 4.1.3 (<https://www.r-project.org/>) within RStudio software version 1.4.1106 (<https://www.rstudio.com/>) using relevant packages by a reviewer (JYZ) under supervision of a statistical expert (JJL). All of the statistical tests were 2-sided. The alpha level for statistically significance is set at 0.05, if not stated otherwise.

The continuous variables will present as mean ± standard deviation (SD) if they showing a normal distribution, or median (interquartile range, IQR) if they showing a non-normal distribution. The categorical variables will present as frequency distribution (n) and percentages (%). To assess intergroup differences, the independent sample t test was used for continuous variables showing a normal distribution, the Mann–Whitney U test for non-normally distributed variables. The chi-square test for categorical variables, and Fisher’s exact test for those with small sample sizes. To assess multigroup differences the analysis of variables (ANOVA) will be used for those without heterogeneity of variance, and the Kruskal–Wallis H test will be used for those with heterogeneity of variance.

The univariate and multivariate logistic regression will be used to analyze the potential influencing factors of reporting guidelines endorsement for all 23 reporting guidelines, for 15 general reporting guidelines, and for 8 artificial intelligence reporting guidelines, respectively. Odds ratios (ORs) and 95% confidence intervals (CIs) will be calculated to investigate whether (1) JIF quartile (Q1, Q2, Q3, Q4); (2) publication institution/publisher (Elsevier, Lippincott Williams & Wilkins, Springer, Wiley, Oxford, etc.); (3) region (North America, Europe, Asia); (4) publication frequency (monthly, bi-monthly, quarterly, etc.); (5) type of access (conventional, open, or hybrid); (6) Only in Radiology, Nuclear Medicine & Medical Imaging category (yes, no); (7) official journal owned by societies (yes, no), are associated with the reporting guidelines endorsement. The alpha level for univariate logistic regression will set at 0.10. Multiple logistic regression analysis will be used to estimate adjusted ORs and 95% CIs of whether factors are associated with the reporting guidelines endorsement. The alpha level for multivariate logistic regression will set at 0.05.

**[Modification]**

The univariate and multivariate logistic regression will be only performed for the overall endorsement of reporting guidelines, but not for the general reporting guidelines or artificial intelligence reporting guidelines. It will be of nonsense, since there is extremely low endorsement of artificial intelligence reporting guidelines. The factors will be included into the multivariate logistic regression if the factor is considered to be potentially associated with the reporting guidelines endorsement in the univariate logistic regression.

**Reporting and dissemination**

We plan to report this study via peer-reviewed journals. One reviewer will draft the original version of the manuscript. All the reviewers will read and edit the manuscript critically. We plan to disseminate our study via conference abstracts, journal articles, and oral presentations.

**Reference**

1. Turner L, Shamseer L, Altman DG, Schulz KF, Moher D (2012) Does use of the CONSORT Statement impact the completeness of reporting of randomised controlled trials published in medical journals? A Cochrane review. Syst Rev 1:60. <https://doi.org/10.1186/2046-4053-1-60>
2. Korevaar DA, van Enst WA, Spijker R, Bossuyt PM, Hooft L (2014) Reporting quality of diagnostic accuracy studies: a systematic review and meta-analysis of investigations on adherence to STARD. Evid Based Med 19(2):47-54. <https://doi.org/10.1136/eb-2013-101637>
3. Stevens A, Shamseer L, Weinstein E et al (2014) Relation of completeness of reporting of health research to journals' endorsement of reporting guidelines: systematic review. BMJ 348:g3804. <https://doi.org/10.1136/bmj.g3804>
4. Stahl AC, Tietz AS, Dewey M, Kendziora B (2023) Has the quality of reporting improved since it became mandatory to use the Standards for Reporting Diagnostic Accuracy? Insights Imaging 14(1):85. <https://doi.org/10.1186/s13244-023-01432-7>
5. Stahl AC, Tietz AS, Kendziora B, Dewey M (2023) Has the STARD statement improved the quality of reporting of diagnostic accuracy studies published in European Radiology? Eur Radiol 33(1):97-105. <https://doi.org/10.1007/s00330-022-09008-7>
6. Dewey M, Levine D, Bossuyt PM, Kressel HY (2019) Impact and perceived value of journal reporting guidelines among Radiology authors and reviewers. Eur Radiol 29(8):3986-3995. <https://doi.org/10.1007/s00330-018-5980-3>
7. Schulz KF, Altman DG, Moher D; CONSORT Group (2010) CONSORT 2010 statement: updated guidelines for reporting parallel group randomized trials. Ann Intern Med 152(11):726-32. <https://doi.org/10.7326/0003-4819-152-11-201006010-00232>
8. von Elm E, Altman DG, Egger M, Pocock SJ, Gøtzsche PC, Vandenbroucke JP; STROBE Initiative (2007) The Strengthening the Reporting of Observational Studies in Epidemiology (STROBE) statement: guidelines for reporting observational studies. Ann Intern Med 147(8):573-7. <https://doi.org/10.7326/0003-4819-147-8-200710160-00010>
9. Page MJ, McKenzie JE, Bossuyt PM et al (2021) The PRISMA 2020 statement: an updated guideline for reporting systematic reviews. BMJ 372:n71. <https://doi.org/10.1136/bmj.n71>
10. Chan AW, Tetzlaff JM, Altman DG et al (2013) SPIRIT 2013 statement: defining standard protocol items for clinical trials. Ann Intern Med 158(3):200-7. <https://doi.org/10.7326/0003-4819-158-3-201302050-00583>
11. Moher D, Shamseer L, Clarke M et al; PRISMA-P Group (2015) Preferred reporting items for systematic review and meta-analysis protocols (PRISMA-P) 2015 statement. Syst Rev 4(1):1. <https://doi.org/10.1186/2046-4053-4-1>
12. Bossuyt PM, Reitsma JB, Bruns DE et al; STARD Group (2015) STARD 2015: An Updated List of Essential Items for Reporting Diagnostic Accuracy Studies. Radiology 277(3):826-32. <https://doi.org/10.1148/radiol.2015151516>
13. Collins GS, Reitsma JB, Altman DG, Moons KG (2015) Transparent Reporting of a multivariable prediction model for Individual Prognosis or Diagnosis (TRIPOD): the TRIPOD statement. Ann Intern Med 162(1):55-63. <https://doi.org/10.7326/M14-0697>
14. Gagnier JJ, Kienle G, Altman DG, Moher D, Sox H, Riley D; CARE Group (2013) The CARE guidelines: consensus-based clinical case reporting guideline development. BMJ Case Rep 2013:bcr2013201554. <https://doi.org/10.1136/bcr-2013-201554>
15. Brouwers MC, Kerkvliet K, Spithoff K; AGREE Next Steps Consortium (2016) The AGREE reporting checklist: a tool to improve reporting of clinical practice guidelines. BMJ 352:i1152. <https://doi.org/.org/10.1136/bmj.i1152>
16. Chen Y, Yang K, Marušic A et al; RIGHT (Reporting Items for Practice Guidelines in Healthcare) Working Group (2017) A reporting tool for practice guidelines in health care: the RIGHT statement. Ann Intern Med 166(2):128-132. <https://doi.org/10.7326/M16-1565>
17. O'Brien BC, Harris IB, Beckman TJ, Reed DA, Cook DA (2014) Standards for reporting qualitative research: a synthesis of recommendations. Acad Med 89(9):1245-51. <https://doi.org/10.1097/ACM.0000000000000388>
18. Tong A, Sainsbury P, Craig J (2007) Consolidated criteria for reporting qualitative research (COREQ): a 32-item checklist for interviews and focus groups. Int J Qual Health Care 19(6):349-57. <https://doi.org/10.1093/intqhc/mzm042>
19. Percie du Sert N, Hurst V et al (2020) The ARRIVE guidelines 2.0: Updated guidelines for reporting animal research. PLoS Biol 18(7):e3000410. <https://doi.org/10.1371/journal.pbio.3000410>
20. Ogrinc G, Davies L, Goodman D, Batalden P, Davidoff F, Stevens D (2016) SQUIRE 2.0 (Standards for QUality Improvement Reporting Excellence): revised publication guidelines from a detailed consensus process. BMJ Qual Saf 25(12):986-992. <https://doi.org/10.1136/bmjqs-2015-004411>
21. Husereau D, Drummond M, Augustovski F et al; CHEERS 2022 ISPOR Good Research Practices Task Force (2022) Consolidated Health Economic Evaluation Reporting Standards 2022 (CHEERS 2022) statement: updated reporting guidance for health economic evaluations. BMJ 376:e067975. <https://doi.org/10.1136/bmj-2021-067975>
22. Kunath F, Grobe HR, Rücker G et al (2012) Do journals publishing in the field of urology endorse reporting guidelines? A survey of author instructions. Urol Int 88(1):54-9. <https://doi.org/10.1159/000332742>
23. Sims MT, Henning NM, Wayant CC, Vassar M (2016) Do emergency medicine journals promote trial registration and adherence to reporting guidelines? A survey of "Instructions for Authors". Scand J Trauma Resusc Emerg Med 24(1):137. <https://doi.org/10.1186/s13049-016-0331-3>
24. Wayant C, Smith C, Sims M, Vassar M (2017) Hematology journals do not sufficiently adhere to reporting guidelines: a systematic review. J Thromb Haemost 15(4):608-617. <https://doi.org/10.1111/jth.13637>
25. Checketts JX, Sims MT, Detweiler B, Middlemist K, Jones J, Vassar M (2018) An evaluation of reporting guidelines and clinical trial registry requirements among orthopaedic surgery journals. J Bone Joint Surg Am 100(3):e15. <https://doi.org/10.2106/JBJS.17.00529>
26. Sims MT, Checketts JX, Wayant C, Vassar M (2018) Requirements for trial registration and adherence to reporting guidelines in critical care journals: a meta-epidemiological study of journals' instructions for authors. Int J Evid Based Healthc 16(1):55-65. <https://doi.org/10.1097/XEB.0000000000000120>
27. Sims MT, Bowers AM, Fernan JM, Dormire KD, Herrington JM, Vassar M (2018) Trial registration and adherence to reporting guidelines in cardiovascular journals. Heart 104(9):753-759. <https://doi.org/10.1136/heartjnl-2017-312165>
28. Jorski A, Scott J, Heavener T, Vassar M (2018) Reporting guideline and clinical trial registration requirements in gastroenterology and hepatology journals. Int J Evid Based Healthc 16(2):119-127. <https://doi.org/10.1097/XEB.0000000000000135>
29. Cook C, Checketts JX, Atakpo P, Nelson N, Vassar M (2018) How well are reporting guidelines and trial registration used by dermatology journals to limit bias? A meta-epidemiological study. Br J Dermatol 178(6):1433-1434. <https://doi.org/10.1111/bjd.16135>
30. Wayant C, Moore G, Hoelscher M, Cook C, Vassar M (2018) Adherence to reporting guidelines and clinical trial registration policies in oncology journals: a cross-sectional review. BMJ Evid Based Med 23(3):104-110. <https://doi.org/10.1136/bmjebm-2017-110855>
31. Sharp MK, Tokalić R, Gómez G, Wager E, Altman DG, Hren D (2019) A cross-sectional bibliometric study showed suboptimal journal endorsement rates of STROBE and its extensions. J Clin Epidemiol 107:42-50. <https://doi.org/10.1016/j.jclinepi.2018.11.006>
32. Zuñiga-Hernandez JA, Dorsey-Treviño EG, González-González JG, Brito JP, Montori VM, Rodriguez-Gutierrez R (2019) Endorsement of reporting guidelines and study registration by endocrine and internal medicine journals: meta-epidemiological study. BMJ Open 9(9):e031259. <https://doi.org/10.1136/bmjopen-2019-031259>
33. Zhou J, Li J, Zhang J, Geng B, Chen Y, Zhou X (2021) Requirements for study registration and adherence to reporting guidelines in surgery journals: a cross-sectional study. World J Surg 45(4):1031-1042. <https://doi.org/10.1007/s00268-020-05920-5>
34. Zhou J, Li J, Zhang J, Geng B, Chen Y, Zhou X (2022) The relationship between endorsing reporting guidelines or trial registration and the impact factor or total citations in surgical journals. PeerJ 10:e12837. <https://doi.org/10.7717/peerj.12837>
35. Duan Y, Zhao L, Ma Y et al (2023) A cross-sectional study of the endorsement proportion of reporting guidelines in 1039 Chinese medical journals. BMC Med Res Methodol 23(1):20. <https://doi.org/10.1186/s12874-022-01789-1>
36. Liu X, Cruz Rivera S, Moher D, Calvert MJ, Denniston AK; SPIRIT-AI and CONSORT-AI Working Group (2020) Reporting guidelines for clinical trial reports for interventions involving artificial intelligence: the CONSORT-AI extension. Lancet Digit Health 2(10):e537-e548. <https://doi.org/10.1016/S2589-7500(20)30218-1>
37. Cruz Rivera S, Liu X, Chan AW, Denniston AK, Calvert MJ; SPIRIT-AI and CONSORT-AI Working Group (2020) Guidelines for clinical trial protocols for interventions involving artificial intelligence: the SPIRIT-AI extension. Lancet Digit Health 2(10):e549-e560. <https://doi.org/10.1016/S2589-7500(20)30219-3>
38. Lekadir K, Osuala R, Gallin C et al (2021) FUTURE-AI: Guiding principles and consensus recommendations for trustworthy artificial intelligence in medical imaging. arXiv 2109.09658v3. <https://arxiv.org/abs/2109.09658> Accessed 15 July 2023.
39. Norgeot B, Quer G, Beaulieu-Jones BK et al (2020) Minimum information about clinical artificial intelligence modeling: the MI-CLAIM checklist. Nat Med 26(9):1320-1324. <https://doi.org/10.1038/s41591-020-1041-y>
40. Hernandez-Boussard T, Bozkurt S, Ioannidis JPA, Shah NH (2020) MINIMAR (MINimum Information for Medical AI Reporting): Developing reporting standards for artificial intelligence in health care. J Am Med Inform Assoc 27(12):2011-2015. <https://doi.org/10.1093/jamia/ocaa088>
41. Mongan J, Moy L, Kahn CE Jr (2020) Checklist for Artificial Intelligence in Medical Imaging (CLAIM): a guide for authors and reviewers. Radiol Artif Intell 2(2):e200029. <https://doi.org/10.1148/ryai.2020200029>
42. Cerdá-Alberich L, Solana J, Mallol P et al (2023) MAIC-10 brief quality checklist for publications using artificial intelligence and medical images. Insights Imaging 14(1):11. <https://doi.org/10.1186/s13244-022-01355-9>
43. Lambin P, Leijenaar RTH, Deist TM et al (2017) Radiomics: the bridge between medical imaging and personalized medicine. Nat Rev Clin Oncol 14(12):749-762. <https://doi.org/10.1038/nrclinonc.2017.141>
44. Zwanenburg A, Vallières M, Abdalah MA et al (2020) The image biomarker standardization initiative: standardized quantitative radiomics for high-throughput image-based phenotyping. Radiology 295(2):328-338. <https://doi.org/10.1148/radiol.2020191145>
45. Kocak B, Baessler B, Bakas S et al (2023) CheckList for EvaluAtion of Radiomics research (CLEAR): a step-by-step reporting guideline for authors and reviewers endorsed by ESR and EuSoMII. Insights Imaging 14(1):75. <https://doi.org/10.1186/s13244-023-01415-8>
46. Ibrahim H, Liu X, Denniston AK (2021) Reporting guidelines for artificial intelligence in healthcare research. Clin Exp Ophthalmol 49(5):470-476. <https://doi.org/10.1111/ceo.13943>
47. Shelmerdine SC, Arthurs OJ, Denniston A, Sebire NJ (2021) Review of study reporting guidelines for clinical studies using artificial intelligence in healthcare. BMJ Health Care Inform 28(1):e100385. <https://doi.org/10.1136/bmjhci-2021-100385>
48. Zrubka Z, Gulácsi L, Péntek M (2022) Time to start using checklists for reporting artificial intelligence in health care and biomedical research: a rapid review of available tools. 2022 IEEE 26th International Conference on Intelligent Engineering System. August 12-15, 2022. Crete, Greece. <https://doi.org/10.1109/INES56734.2022.9922639>
49. Klontzas ME, Gatti AA, Tejani AS, Kahn CE Jr (2023) AI reporting guidelines: How to select the best one for your research. Radiol Artif Intell 5(3):e230055. <https://doi.org/10.1148/ryai.230055>

**Supplementary Table S1 Bibliometrics information of screened journals**

| **No** | **Journal** | **JCR Abbreviation** | **ISSN** | **eISSN** | **2022 JIF** | **JIF Quartile** | **JIF Rank** | **Total Citations** | **Citable Items** |
| --- | --- | --- | --- | --- | --- | --- | --- | --- | --- |
| **1** | RADIOLOGY | RADIOLOGY | 0033-8419 | N/A | 19.7 | Q1 | 1/135 | 64,735 | 265 |
| **2** | JACC-Cardiovascular Imaging | JACC-CARDIOVASC IMAG | 1936-878X | 1876-7591 | 14.0 | Q1 | 2/135 | 16,914 | 115 |
| **3** | MEDICAL IMAGE ANALYSIS | MED IMAGE ANAL | 1361-8415 | 1361-8423 | 10.9 | Q1 | 3/135 | 19,500 | 274 |
| **4** | CLINICAL NUCLEAR MEDICINE | CLIN NUCL MED | 0363-9762 | 1536-0229 | 10.6 | Q1 | 4/135 | 6,883 | 84 |
| **5** | IEEE TRANSACTIONS ON MEDICAL IMAGING | IEEE T MED IMAGING | 0278-0062 | 1558-254X | 10.6 | Q1 | 4/135 | 34,640 | 319 |
| **6** | JOURNAL OF NUCLEAR MEDICINE | J NUCL MED | 0161-5505 | 1535-5667 | 9.3 | Q1 | 6/135 | 31,896 | 266 |
| **7** | EUROPEAN JOURNAL OF NUCLEAR MEDICINE AND MOLECULAR IMAGING | EUR J NUCL MED MOL I | 1619-7070 | 1619-7089 | 9.1 | Q1 | 7/135 | 23,232 | 318 |
| **8** | Radiologia Medica | RADIOL MED | 0033-8362 | 1826-6983 | 8.9 | Q1 | 8/135 | 5,396 | 136 |
| **9** | Photoacoustics | PHOTOACOUSTICS | 2213-5979 | 2213-5979 | 7.9 | Q1 | 9/135 | 2,551 | 94 |
| **10** | Circulation-Cardiovascular Imaging | CIRC-CARDIOVASC IMAG | 1941-9651 | 1942-0080 | 7.5 | Q1 | 10/135 | 8,076 | 53 |
| **11** | ULTRASOUND IN OBSTETRICS & GYNECOLOGY | ULTRASOUND OBST GYN | 0960-7692 | 1469-0705 | 7.1 | Q1 | 11/135 | 17,581 | 160 |
| **12** | INTERNATIONAL JOURNAL OF RADIATION ONCOLOGY BIOLOGY PHYSICS | INT J RADIAT ONCOL | 0360-3016 | 1879-355X | 7.0 | Q1 | 12/135 | 46,275 | 280 |
| **13** | INVESTIGATIVE RADIOLOGY | INVEST RADIOL | 0020-9996 | 1536-0210 | 6.7 | Q1 | 13/135 | 6,838 | 87 |
| **14** | JOURNAL OF CARDIOVASCULAR MAGNETIC RESONANCE | J CARDIOVASC MAGN R | 1097-6647 | 1532-429X | 6.4 | Q1 | 14/135 | 7,533 | 71 |
| **15** | European Heart Journal-Cardiovascular Imaging | EUR HEART J-CARD IMG | 2047-2404 | 2047-2412 | 6.2 | Q1 | 15/135 | 10,828 | 147 |
| **16** | EUROPEAN RADIOLOGY | EUR RADIOL | 0938-7994 | 1432-1084 | 5.9 | Q1 | 16/135 | 34,829 | 902 |
| **17** | COMPUTERIZED MEDICAL IMAGING AND GRAPHICS | COMPUT MED IMAG GRAP | 0895-6111 | 1879-0771 | 5.7 | Q1 | 17/135 | 4,576 | 81 |
| **18** | NEUROIMAGE | NEUROIMAGE | 1053-8119 | 1095-9572 | 5.7 | Q1 | 17/135 | 120,835 | 760 |
| **19** | RADIOTHERAPY AND ONCOLOGY | RADIOTHER ONCOL | 0167-8140 | 1879-0887 | 5.7 | Q1 | 17/135 | 23,221 | 351 |
| **20** | Diagnostic and Interventional Imaging | DIAGN INTERV IMAG | 2211-5684 | 2211-5684 | 5.5 | Q1 | 20/135 | 3,017 | 66 |
| **21** | RADIOGRAPHICS | RADIOGRAPHICS | 0271-5333 | N/A | 5.5 | Q1 | 20/135 | 17,226 | 125 |
| **22** | Journal of Cardiovascular Computed Tomography | J CARDIOVASC COMPUT | 1934-5925 | 1934-5925 | 5.4 | Q1 | 22/135 | 2,968 | 56 |
| **23** | AMERICAN JOURNAL OF ROENTGENOLOGY | AM J ROENTGENOL | 0361-803X | 1546-3141 | 5.0 | Q1 | 23/135 | 35,480 | 178 |
| **24** | CANCER IMAGING | CANCER IMAGING | 1740-5025 | 1470-7330 | 4.9 | Q1 | 24/135 | 2,779 | 76 |
| **25** | SEMINARS IN NUCLEAR MEDICINE | SEMIN NUCL MED | 0001-2998 | 1558-4623 | 4.9 | Q1 | 24/135 | 2,877 | 79 |
| **26** | ACADEMIC RADIOLOGY | ACAD RADIOL | 1076-6332 | 1878-4046 | 4.8 | Q1 | 26/135 | 8,982 | 308 |
| **27** | HUMAN BRAIN MAPPING | HUM BRAIN MAPP | 1065-9471 | 1097-0193 | 4.8 | Q1 | 26/135 | 28,764 | 397 |
| **28** | KOREAN JOURNAL OF RADIOLOGY | KOREAN J RADIOL | 1229-6929 | 2005-8330 | 4.8 | Q1 | 26/135 | 5,377 | 95 |
| **29** | Insights into Imaging | INSIGHTS IMAGING | 1869-4101 | 1869-4101 | 4.7 | Q1 | 29/135 | 5,008 | 194 |
| **30** | Journal of the American College of Radiology | J AM COLL RADIOL | 1546-1440 | 1558-349X | 4.5 | Q1 | 30/135 | 7,214 | 134 |
| **31** | JOURNAL OF DIGITAL IMAGING | J DIGIT IMAGING | 0897-1889 | 1618-727X | 4.4 | Q1 | 31/135 | 5,216 | 207 |
| **32** | JOURNAL OF MAGNETIC RESONANCE IMAGING | J MAGN RESON IMAGING | 1053-1807 | 1522-2586 | 4.4 | Q1 | 31/135 | 20,316 | 317 |
| **33** | Physical and Engineering Sciences in Medicine | PHYS ENG SCI MED | 2662-4729 | 2662-4737 | 4.4 | Q1 | 31/135 | 1,025 | 107 |
| **34** | ULTRASONICS | ULTRASONICS | 0041-624X | 1874-9968 | 4.2 | Q2 | 34/135 | 9,972 | 185 |
| **35** | EJNMMI Physics | EJNMMI PHYS | 2197-7364 | 2197-7364 | 4.0 | Q2 | 35/135 | 1,273 | 88 |
| **36** | MEDICAL PHYSICS | MED PHYS | 0094-2405 | 2473-4209 | 3.8 | Q2 | 36/135 | 31,491 | 658 |
| **37** | Radiation Oncology | RADIAT ONCOL | N/A | 1748-717X | 3.6 | Q2 | 37/135 | 8,845 | 213 |
| **38** | AMERICAN JOURNAL OF NEURORADIOLOGY | AM J NEURORADIOL | 0195-6108 | 1936-959X | 3.5 | Q2 | 38/135 | 25,993 | 272 |
| **39** | JOURNAL OF BIOMEDICAL OPTICS | J BIOMED OPT | 1083-3668 | 1560-2281 | 3.5 | Q2 | 38/135 | 14,729 | 168 |
| **40** | JOURNAL OF NEURORADIOLOGY | J NEURORADIOLOGY | 0150-9861 | 1773-0406 | 3.5 | Q2 | 38/135 | 1,505 | 50 |
| **41** | PHYSICS IN MEDICINE AND BIOLOGY | PHYS MED BIOL | 0031-9155 | 1361-6560 | 3.5 | Q2 | 38/135 | 29,272 | 422 |
| **42** | SEMINARS IN RADIATION ONCOLOGY | SEMIN RADIAT ONCOL | 1053-4296 | 1532-9461 | 3.5 | Q2 | 38/135 | 2,801 | 45 |
| **43** | Biomedical Optics Express | BIOMED OPT EXPRESS | 2156-7085 | 2156-7085 | 3.4 | Q2 | 43/135 | 14,699 | 450 |
| **44** | CANCER BIOTHERAPY AND RADIOPHARMACEUTICALS | CANCER BIOTHER RADIO | 1084-9785 | 1557-8852 | 3.4 | Q2 | 43/135 | 2,857 | 46 |
| **45** | Physica Medica-European Journal of Medical Physics | PHYS MEDICA | 1120-1797 | 1724-191X | 3.4 | Q2 | 43/135 | 5,488 | 200 |
| **46** | RADIATION RESEARCH | RADIAT RES | 0033-7587 | 1938-5404 | 3.4 | Q2 | 43/135 | 9,234 | 109 |
| **47** | ULTRASCHALL IN DER MEDIZIN | ULTRASCHALL MED | 0172-4614 | 1438-8782 | 3.4 | Q2 | 43/135 | 2,686 | 51 |
| **48** | DENTOMAXILLOFACIAL RADIOLOGY | DENTOMAXILLOFAC RAD | 0250-832X | 1476-542X | 3.3 | Q2 | 48/135 | 4,025 | 83 |
| **49** | EUROPEAN JOURNAL OF RADIOLOGY | EUR J RADIOL | 0720-048X | 1872-7727 | 3.3 | Q2 | 48/135 | 17,302 | 410 |
| **50** | JOURNAL OF THORACIC IMAGING | J THORAC IMAG | 0883-5993 | 1536-0237 | 3.3 | Q2 | 48/135 | 2,018 | 61 |
| **51** | MAGNETIC RESONANCE IN MEDICINE | MAGN RESON MED | 0740-3194 | 1522-2594 | 3.3 | Q2 | 48/135 | 32,434 | 383 |
| **52** | Practical Radiation Oncology | PRACT RADIAT ONCOL | 1879-8500 | 1879-8500 | 3.3 | Q2 | 48/135 | 2,793 | 81 |
| **53** | EJNMMI Research | EJNMMI RES | 2191-219X | 2191-219X | 3.2 | Q2 | 53/135 | 2,875 | 77 |
| **54** | CANADIAN ASSOCIATION OF RADIOLOGISTS JOURNAL-JOURNAL DE L ASSOCIATION CANADIENNE DES RADIOLOGISTES | CAN ASSOC RADIOL J | 0846-5371 | 1488-2361 | 3.1 | Q2 | 54/135 | 1,513 | 62 |
| **55** | Clinical and Translational Radiation Oncology | CLIN TRANSL RAD ONCO | N/A | 2405-6308 | 3.1 | Q2 | 54/135 | 1,540 | 128 |
| **56** | INTERNATIONAL JOURNAL OF HYPERTHERMIA | INT J HYPERTHER | 0265-6736 | 1464-5157 | 3.1 | Q2 | 54/135 | 6,456 | 153 |
| **57** | MOLECULAR IMAGING AND BIOLOGY | MOL IMAGING BIOL | 1536-1632 | 1860-2002 | 3.1 | Q2 | 54/135 | 3,530 | 94 |
| **58** | NUCLEAR MEDICINE AND BIOLOGY | NUCL MED BIOL | 0969-8051 | 1872-9614 | 3.1 | Q2 | 54/135 | 3,714 | 59 |
| **59** | STRAHLENTHERAPIE UND ONKOLOGIE | STRAHLENTHER ONKOL | 0179-7158 | 1439-099X | 3.1 | Q2 | 54/135 | 3,580 | 108 |
| **60** | Ultrasonography | ULTRASONOGRAPHY | 2288-5919 | 2288-5943 | 3.1 | Q2 | 54/135 | 1,202 | 69 |
| **61** | International Journal of Computer Assisted Radiology and Surgery | INT J COMPUT ASS RAD | 1861-6410 | 1861-6429 | 3.0 | Q2 | 61/135 | 5,458 | 264 |
| **62** | Magnetic Resonance in Medical Sciences | MAGN RESON MED SCI | 1347-3182 | 1880-2206 | 3.0 | Q2 | 61/135 | 1,292 | 51 |
| **63** | CARDIOVASCULAR AND INTERVENTIONAL RADIOLOGY | CARDIOVASC INTER RAD | 0174-1551 | 1432-086X | 2.9 | Q2 | 63/135 | 7,246 | 185 |
| **64** | JOURNAL OF VASCULAR AND INTERVENTIONAL RADIOLOGY | J VASC INTERV RADIOL | 1051-0443 | 1535-7732 | 2.9 | Q2 | 63/135 | 10,463 | 161 |
| **65** | NMR IN BIOMEDICINE | NMR BIOMED | 0952-3480 | 1099-1492 | 2.9 | Q2 | 63/135 | 8,316 | 201 |
| **66** | ULTRASOUND IN MEDICINE AND BIOLOGY | ULTRASOUND MED BIOL | 0301-5629 | 1879-291X | 2.9 | Q2 | 63/135 | 13,059 | 229 |
| **67** | Clinical Neuroradiology | CLIN NEURORADIOL | 1869-1439 | 1869-1447 | 2.8 | Q2 | 67/135 | 1,788 | 99 |
| **68** | Molecular Imaging | MOL IMAGING | N/A | 1536-0121 | 2.8 | Q2 | 67/135 | 1,175 | 21 |
| **69** | NEURORADIOLOGY | NEURORADIOLOGY | 0028-3940 | 1432-1920 | 2.8 | Q2 | 67/135 | 7,101 | 194 |
| **70** | Quantitative Imaging in Medicine and Surgery | QUANT IMAG MED SURG | 2223-4292 | 2223-4306 | 2.8 | Q2 | 67/135 | 4,218 | 331 |
| **71** | BMC MEDICAL IMAGING | BMC MED IMAGING | 1471-2342 | 1471-2342 | 2.7 | Q3 | 71/135 | 2,624 | 224 |
| **72** | ANNALS OF NUCLEAR MEDICINE | ANN NUCL MED | 0914-7187 | 1864-6433 | 2.6 | Q3 | 72/135 | 2,968 | 107 |
| **73** | BRITISH JOURNAL OF RADIOLOGY | BRIT J RADIOL | 0007-1285 | 1748-880X | 2.6 | Q3 | 72/135 | 13,683 | 239 |
| **74** | CLINICAL RADIOLOGY | CLIN RADIOL | 0009-9260 | 1365-229X | 2.6 | Q3 | 72/135 | 8,265 | 214 |
| **75** | INTERNATIONAL JOURNAL OF RADIATION BIOLOGY | INT J RADIAT BIOL | 0955-3002 | 1362-3095 | 2.6 | Q3 | 72/135 | 6,356 | 146 |
| **76** | Dose-Response | DOSE-RESPONSE | 1559-3258 | 1559-3258 | 2.5 | Q3 | 76/135 | 2,226 | 128 |
| **77** | Journal of Innovative Optical Health Sciences | J INNOV OPT HEAL SCI | 1793-5458 | 1793-7205 | 2.5 | Q3 | 76/135 | 972 | 84 |
| **78** | MAGNETIC RESONANCE IMAGING | MAGN RESON IMAGING | 0730-725X | 1873-5894 | 2.5 | Q3 | 76/135 | 8,503 | 161 |
| **79** | Abdominal Radiology | ABDOM RADIOL | 2366-004X | 2366-0058 | 2.4 | Q3 | 79/135 | 6,231 | 344 |
| **80** | JOURNAL OF NEUROIMAGING | J NEUROIMAGING | 1051-2284 | 1552-6569 | 2.4 | Q3 | 79/135 | 3,005 | 109 |
| **81** | JOURNAL OF NUCLEAR CARDIOLOGY | J NUCL CARDIOL | 1071-3581 | 1532-6551 | 2.4 | Q3 | 79/135 | 4,195 | 125 |
| **82** | Radiology and Oncology | RADIOL ONCOL | 1318-2099 | 1581-3207 | 2.4 | Q3 | 79/135 | 1,500 | 52 |
| **83** | Current Radiopharmaceuticals | CURR RADIOPHARM | 1874-4710 | 1874-4729 | 2.3 | Q3 | 83/135 | 581 | 35 |
| **84** | JOURNAL OF ULTRASOUND IN MEDICINE | J ULTRAS MED | 0278-4297 | 1550-9613 | 2.3 | Q3 | 83/135 | 9,142 | 185 |
| **85** | MAGNETIC RESONANCE MATERIALS IN PHYSICS BIOLOGY AND MEDICINE | MAGN RESON MATER PHY | 1352-8661 | 1352-8661 | 2.3 | Q3 | 83/135 | 1,828 | 59 |
| **86** | NEUROIMAGING CLINICS OF NORTH AMERICA | NEUROIMAG CLIN N AM | 1052-5149 | 1557-9867 | 2.3 | Q3 | 83/135 | 1,649 | 39 |
| **87** | PEDIATRIC RADIOLOGY | PEDIATR RADIOL | 0301-0449 | 1432-1998 | 2.3 | Q3 | 83/135 | 8,169 | 248 |
| **88** | ULTRASONIC IMAGING | ULTRASONIC IMAGING | 0161-7346 | 1096-0910 | 2.3 | Q3 | 83/135 | 1,072 | 16 |
| **89** | HEALTH PHYSICS | HEALTH PHYS | 0017-9078 | 1538-5159 | 2.2 | Q3 | 89/135 | 4,666 | 56 |
| **90** | Clinical and Translational Imaging | CLIN TRANSL IMAGING | 2281-5872 | 2281-7565 | 2.1 | Q3 | 90/135 | 713 | 50 |
| **91** | Clinical Imaging | CLIN IMAG | 0899-7071 | 1873-4499 | 2.1 | Q3 | 90/135 | 4,348 | 191 |
| **92** | Diagnostic and Interventional Radiology | DIAGN INTERV RADIOL | N/A | 1305-3612 | 2.1 | Q3 | 90/135 | 2,043 | 84 |
| **93** | INTERNATIONAL JOURNAL OF CARDIOVASCULAR IMAGING | INT J CARDIOVAS IMAG | 1569-5794 | 1875-8312 | 2.1 | Q3 | 90/135 | 4,864 | 227 |
| **94** | Japanese Journal of Radiology | JPN J RADIOL | 1867-1071 | 1867-108X | 2.1 | Q3 | 90/135 | 2,064 | 128 |
| **95** | Journal of Applied Clinical Medical Physics | J APPL CLIN MED PHYS | 1526-9914 | 1526-9914 | 2.1 | Q3 | 90/135 | 4,930 | 337 |
| **96** | SKELETAL RADIOLOGY | SKELETAL RADIOL | 0364-2348 | 1432-2161 | 2.1 | Q3 | 90/135 | 7,266 | 223 |
| **97** | JOURNAL OF RADIATION RESEARCH | J RADIAT RES | 0449-3060 | 1349-9157 | 2.0 | Q3 | 97/135 | 3,576 | 91 |
| **98** | Journal of the Belgian Society of Radiology | J BELG SOC RADIOL | 2514-8281 | 2514-8281 | 2.0 | Q3 | 97/135 | 311 | 56 |
| **99** | Zeitschrift fur Medizinische Physik | Z MED PHYS | 0939-3889 | 1876-4436 | 2.0 | Q3 | 97/135 | 1,007 | 37 |
| **100** | Brachytherapy | BRACHYTHERAPY | 1538-4721 | 1873-1449 | 1.9 | Q3 | 100/135 | 2,455 | 102 |
| **101** | RADIOLOGIC CLINICS OF NORTH AMERICA | RADIOL CLIN N AM | 0033-8389 | 1557-8275 | 1.9 | Q3 | 100/135 | 2,676 | 73 |
| **102** | Tomography | TOMOGRAPHY | 2379-1381 | 2379-139X | 1.9 | Q3 | 100/135 | 810 | 241 |
| **103** | Journal of Medical Ultrasonics | J MED ULTRASON | 1346-4523 | 1613-2254 | 1.8 | Q4 | 103/135 | 850 | 69 |
| **104** | ROFO-FORTSCHRITTE AUF DEM GEBIET DER RONTGENSTRAHLEN UND DER BILDGEBENDEN VERFAHREN | ROFO-FORTSCHR RONTG | 1438-9029 | 1438-9010 | 1.8 | Q4 | 103/135 | 1,764 | 63 |
| **105** | INTERVENTIONAL NEURORADIOLOGY | INTERV NEURORADIOL | 1591-0199 | 2385-2011 | 1.7 | Q4 | 105/135 | 2,174 | 211 |
| **106** | Medical Ultrasonography | MED ULTRASON | 1844-4172 | 2066-8643 | 1.7 | Q4 | 105/135 | 1,183 | 61 |
| **107** | RADIATION AND ENVIRONMENTAL BIOPHYSICS | RADIAT ENVIRON BIOPH | 0301-634X | 1432-2099 | 1.7 | Q4 | 105/135 | 1,769 | 47 |
| **108** | Applied Radiation and Isotopes | APPL RADIAT ISOTOPES | 0969-8043 | 1872-9800 | 1.6 | Q4 | 108/135 | 9,557 | 417 |
| **109** | Journal of Medical Imaging and Radiation Oncology | J MED IMAG RADIAT ON | 1754-9477 | 1754-9485 | 1.6 | Q4 | 108/135 | 1,937 | 101 |
| **110** | Magnetic Resonance Imaging Clinics of North America | MAGN RESON IMAGING C | 1064-9689 | 1557-9786 | 1.6 | Q4 | 108/135 | 1,225 | 34 |
| **111** | Hellenic Journal of Nuclear Medicine | HELL J NUCL MED | 1108-1430 | 1790-5427 | 1.5 | Q4 | 111/135 | 544 | 40 |
| **112** | JOURNAL OF RADIOLOGICAL PROTECTION | J RADIOL PROT | 0952-4746 | 1361-6498 | 1.5 | Q4 | 111/135 | 1,779 | 132 |
| **113** | NUCLEAR MEDICINE COMMUNICATIONS | NUCL MED COMMUN | 0143-3636 | 1473-5628 | 1.5 | Q4 | 111/135 | 3,355 | 145 |
| **114** | NUKLEARMEDIZIN-NUCLEAR MEDICINE | NUKLEARMED-NUCL MED | 0029-5566 | 2567-6407 | 1.5 | Q4 | 111/135 | 553 | 28 |
| **115** | QUARTERLY JOURNAL OF NUCLEAR MEDICINE AND MOLECULAR IMAGING | Q J NUCL MED MOL IM | 1824-4785 | 1827-1936 | 1.5 | Q4 | 111/135 | 874 | 34 |
| **116** | Current Medical Imaging | CURR MED IMAGING | 1573-4056 | 1875-6603 | 1.4 | Q4 | 116/135 | 1,054 | 188 |
| **117** | Journal of Contemporary Brachytherapy | J CONTEMP BRACHYTHER | 1689-832X | 2081-2841 | 1.4 | Q4 | 116/135 | 881 | 70 |
| **118** | SEMINARS IN INTERVENTIONAL RADIOLOGY | SEMIN INTERVENT RAD | 0739-9529 | 1098-8963 | 1.4 | Q4 | 116/135 | 1,658 | 78 |
| **119** | SEMINARS IN MUSCULOSKELETAL RADIOLOGY | SEMIN MUSCULOSKEL R | 1089-7860 | 1098-898X | 1.4 | Q4 | 116/135 | 1,160 | 63 |
| **120** | SURGICAL AND RADIOLOGIC ANATOMY | SURG RADIOL ANAT | 0930-1038 | 1279-8517 | 1.4 | Q4 | 116/135 | 4,492 | 186 |
| **121** | ACTA RADIOLOGICA | ACTA RADIOL | 0284-1851 | 1600-0455 | 1.3 | Q4 | 121/135 | 5,292 | 194 |
| **122** | Cancer Radiotherapie | CANCER RADIOTHER | 1278-3218 | 1769-6658 | 1.3 | Q4 | 121/135 | 1,316 | 147 |
| **123** | JOURNAL OF COMPUTER ASSISTED TOMOGRAPHY | J COMPUT ASSIST TOMO | 0363-8715 | 1532-3145 | 1.3 | Q4 | 121/135 | 4,812 | 137 |
| **124** | Ultrasound Quarterly | ULTRASOUND Q | 0894-8771 | 1536-0253 | 1.3 | Q4 | 121/135 | 807 | 47 |
| **125** | Medical Dosimetry | MED DOSIM | 0958-3947 | 1873-4022 | 1.2 | Q4 | 125/135 | 999 | 61 |
| **126** | Revista Espanola de Medicina Nuclear e Imagen Molecular | REV ESP MED NUCL IMA | 2253-654X | N/A | 1.2 | Q4 | 125/135 | 502 | 36 |
| **127** | RADIOPROTECTION | RADIOPROTECTION | 0033-8451 | 1769-700X | 1.1 | Q4 | 127/135 | 515 | 37 |
| **128** | SEMINARS IN ULTRASOUND CT AND MRI | SEMIN ULTRASOUND CT | 0887-2171 | 1558-5034 | 1.1 | Q4 | 127/135 | 1,360 | 46 |
| **129** | RADIATION PROTECTION DOSIMETRY | RADIAT PROT DOSIM | 0144-8420 | 1742-3406 | 1.0 | Q4 | 129/135 | 6,514 | 245 |
| **130** | JOURNAL OF CLINICAL ULTRASOUND | J CLIN ULTRASOUND | 0091-2751 | 1097-0096 | 0.9 | Q4 | 130/135 | 2,442 | 181 |
| **131** | Radiologie | RADIOLOGIE | 2731-7048 | 2731-7056 | 0.7 | Q4 | 131/135 | 696 | 112 |
| **132** | CONCEPTS IN MAGNETIC RESONANCE PART A | CONCEPT MAGN RESON A | 1546-6086 | 1552-5023 | 0.6 | Q4 | 132/135 | 482 | 11 |
| **133** | International Journal of Radiation Research | INT J RADIAT RES | 2322-3243 | 2322-3243 | 0.6 | Q4 | 132/135 | 459 | 133 |
| **134** | SEMINARS IN ROENTGENOLOGY | SEMIN ROENTGENOL | 0037-198X | 1558-4658 | 0.4 | Q4 | 134/135 | 424 | 35 |
| **135** | Iranian Journal of Radiology | IRAN J RADIOL | 1735-1065 | 2008-2711 | 0.3 | Q4 | 135/135 | 446 | 48 |

Note: We search and extracted the data from the Radiology, Nuclear Medicine & Medical Imaging category, Science Citation Index Expanded of the 2022 Journal Citation Reports via Clarivate (<https://jcr.clarivate.com/jcr>) on 20 July 2023.

**Supplementary Table S2 Characteristics and homepages of included journals**

| **No** | **Journal** | **Publisher** | **Region** | **Publication frequency** | **Type of access** | **Only in Radiology** | **Official Journal** | **Homepage** |
| --- | --- | --- | --- | --- | --- | --- | --- | --- |
| **1** | RADIOLOGY | Society | North America | ≥12 | Hybird | Yes | Yes | <https://pubs.rsna.org/journal/radiology> |
| **2** | JACC-Cardiovascular Imaging | Elsevier | North America | ≥12 | Hybird | No | Yes | <https://www.jacc.org/journal/imaging> |
| **3** | MEDICAL IMAGE ANALYSIS | Elsevier | Europe | 6～12 | Hybird | No | Yes | <https://www.sciencedirect.com/journal/medical-image-analysis/> |
| **4** | CLINICAL NUCLEAR MEDICINE | Lippincott Williams & Wilkins | North America | ≥12 | Hybird | Yes | Yes | <https://journals.lww.com/nuclearmed/pages/default.aspx> |
| **5** | IEEE TRANSACTIONS ON MEDICAL IMAGING | Society | North America | ≥12 | Hybird | No | Yes | <https://www.embs.org/tmi/> |
| **6** | JOURNAL OF NUCLEAR MEDICINE | Society | North America | ≥12 | Hybird | Yes | Yes | [https://jnm.snmjournals.org](https://jnm.snmjournals.org/) |
| **7** | EUROPEAN JOURNAL OF NUCLEAR MEDICINE AND MOLECULAR IMAGING | Springer | Europe | ≥12 | Hybird | Yes | Yes | <https://www.springer.com/journal/259> |
| **8** | Radiologia Medica | Springer | Europe | ≥12 | Hybird | Yes | Yes | <https://www.springer.com/journal/11547> |
| **9** | Photoacoustics | Elsevier | Europe | <6 | Open | No | No | <https://www.sciencedirect.com/journal/photoacoustics> |
| **10** | Circulation-Cardiovascular Imaging | Lippincott Williams & Wilkins | North America | ≥12 | Hybird | No | Yes | <https://www.ahajournals.org/journal/circimaging> |
| **11** | ULTRASOUND IN OBSTETRICS & GYNECOLOGY | Wiley | North America | ≥12 | Hybird | No | Yes | <https://obgyn.onlinelibrary.wiley.com/journal/14690705/> |
| **12** | INTERNATIONAL JOURNAL OF RADIATION ONCOLOGY BIOLOGY PHYSICS | Elsevier | North America | ≥12 | Hybird | No | Yes | <https://www.sciencedirect.com/journal/international-journal-of-radiation-oncology-biology-physics> |
| **13** | INVESTIGATIVE RADIOLOGY | Lippincott Williams & Wilkins | North America | ≥12 | Hybird | Yes | No | <https://journals.lww.com/investigativeradiology/pages/default.aspx> |
| **14** | JOURNAL OF CARDIOVASCULAR MAGNETIC RESONANCE | Springer | North America | <6 | Open | No | Yes | [https://jcmr-online.biomedcentral.com](https://jcmr-online.biomedcentral.com/) |
| **15** | European Heart Journal-Cardiovascular Imaging | Other | Europe | ≥12 | Hybird | No | Yes | <https://academic.oup.com/ehjcimaging> |
| **16** | EUROPEAN RADIOLOGY | Springer | Europe | ≥12 | Hybird | Yes | Yes | <https://www.springer.com/journal/330> |
| **17** | COMPUTERIZED MEDICAL IMAGING AND GRAPHICS | Elsevier | North America | 6～12 | Hybird | No | No | <https://www.sciencedirect.com/journal/computerized-medical-imaging-and-graphics/> |
| **18** | NEUROIMAGE | Elsevier | North America | ≥12 | Open | No | No | <https://www.sciencedirect.com/journal/neuroimage> |
| **19** | RADIOTHERAPY AND ONCOLOGY | Elsevier | Europe | ≥12 | Hybird | No | Yes | [https://www.thegreenjournal.com](https://www.thegreenjournal.com/) |
| **20** | Diagnostic and Interventional Imaging | Elsevier | Europe | ≥12 | Open | Yes | Yes | <https://www.sciencedirect.com/journal/diagnostic-and-interventional-imaging/> |
| **21** | Journal of Cardiovascular Computed Tomography | Elsevier | North America | 6～12 | Hybird | No | Yes | [https://www.journalofcardiovascularct.com](https://www.journalofcardiovascularct.com/) |
| **22** | AMERICAN JOURNAL OF ROENTGENOLOGY | Society | North America | ≥12 | Open | Yes | Yes | [https://ajronline.org](https://ajronline.org/) |
| **23** | CANCER IMAGING | Springer | Europe | <6 | Open | No | Yes | [https://cancerimagingjournal.biomedcentral.com](https://cancerimagingjournal.biomedcentral.com/) |
| **24** | ACADEMIC RADIOLOGY | Elsevier | North America | ≥12 | Hybird | Yes | Yes | <https://www.sciencedirect.com/journal/academic-radiology> |
| **25** | HUMAN BRAIN MAPPING | Wiley | North America | ≥12 | Open | No | No | <https://onlinelibrary.wiley.com/journal/10970193> |
| **26** | KOREAN JOURNAL OF RADIOLOGY | Society | Aisa | ≥12 | Open | Yes | Yes | [https://www.kjronline.org](https://www.kjronline.org/) |
| **27** | Insights into Imaging | Springer | Europe | <6 | Open | Yes | Yes | [https://insightsimaging.springeropen.com](https://insightsimaging.springeropen.com/) |
| **28** | Journal of the American College of Radiology | Elsevier | North America | ≥12 | Hybird | Yes | Yes | [https://www.jacr.org](https://www.jacr.org/) |
| **29** | JOURNAL OF DIGITAL IMAGING | Springer | North America | 6～12 | Hybird | Yes | Yes | <https://www.springer.com/journal/10278> |
| **30** | JOURNAL OF MAGNETIC RESONANCE IMAGING | Wiley | North America | ≥12 | Hybird | Yes | Yes | <https://onlinelibrary.wiley.com/journal/15222586?journalRedirectCheck=true> |
| **31** | Physical and Engineering Sciences in Medicine | Springer | Europe | <6 | Hybird | No | Yes | <https://www.springer.com/journal/13246> |
| **32** | ULTRASONICS | Elsevier | Europe | 6～12 | Hybird | No | No | <https://www.sciencedirect.com/journal/ultrasonics> |
| **33** | EJNMMI Physics | Springer | Europe | <6 | Open | Yes | Yes | [https://ejnmmiphys.springeropen.com](https://ejnmmiphys.springeropen.com/) |
| **34** | MEDICAL PHYSICS | Wiley | North America | ≥12 | Hybird | Yes | Yes | [https://www.medphys.org](https://www.medphys.org/) |
| **35** | Radiation Oncology | Springer | Europe | <6 | Open | No | No | [https://ro-journal.biomedcentral.com](https://ro-journal.biomedcentral.com/) |
| **36** | AMERICAN JOURNAL OF NEURORADIOLOGY | Society | North America | ≥12 | Hybird | No | Yes | [https://www.ajnr.org](https://www.ajnr.org/) |
| **37** | JOURNAL OF BIOMEDICAL OPTICS | Society | North America | ≥12 | Open | No | Yes | <https://www.spiedigitallibrary.org/journals/journal-of-biomedical-optics?SSO=1> |
| **38** | JOURNAL OF NEURORADIOLOGY | Elsevier | Europe | <6 | Hybird | No | Yes | <https://www.sciencedirect.com/journal/journal-of-neuroradiology> |
| **39** | PHYSICS IN MEDICINE AND BIOLOGY | Other | Europe | ≥12 | Hybird | No | Yes | <https://iopscience.iop.org/journal/0031-9155> |
| **40** | Biomedical Optics Express | Other | North America | ≥12 | Open | No | No | <https://opg.optica.org/boe/home.cfm> |
| **41** | CANCER BIOTHERAPY AND RADIOPHARMACEUTICALS | Other | North America | 6～12 | Hybird | No | No | <https://home.liebertpub.com/publications/cancer-biotherapy-and-radiopharmaceuticals/8/> |
| **42** | Physica Medica-European Journal of Medical Physics | Elsevier | Europe | 6～12 | Hybird | Yes | Yes | [https://www.physicamedica.com](https://www.physicamedica.com/) |
| **43** | RADIATION RESEARCH | Society | North America | ≥12 | Hybird | No | Yes | <https://meridian.allenpress.com/radiation-research> |
| **44** | DENTOMAXILLOFACIAL RADIOLOGY | Society | Europe | 6～12 | Hybird | No | Yes | <https://www.birpublications.org/journal/dmfr> |
| **45** | EUROPEAN JOURNAL OF RADIOLOGY | Elsevier | Europe | ≥12 | Hybird | Yes | No | <https://www.sciencedirect.com/journal/european-journal-of-radiology> |
| **46** | JOURNAL OF THORACIC IMAGING | Lippincott Williams & Wilkins | North America | 6～12 | Hybird | Yes | Yes | <https://journals.lww.com/thoracicimaging/pages/default.aspx> |
| **47** | MAGNETIC RESONANCE IN MEDICINE | Wiley | North America | ≥12 | Hybird | Yes | Yes | <https://onlinelibrary.wiley.com/journal/15222594> |
| **48** | Practical Radiation Oncology | Elsevier | North America | 6～12 | Hybird | No | Yes | [https://www.practicalradonc.org](https://www.practicalradonc.org/) |
| **49** | EJNMMI Research | Springer | Europe | <6 | Open | Yes | Yes | [https://ejnmmires.springeropen.com](https://ejnmmires.springeropen.com/) |
| **50** | CANADIAN ASSOCIATION OF RADIOLOGISTS JOURNAL-JOURNAL DE L ASSOCIATION CANADIENNE DES RADIOLOGISTES | Other | North America | <6 | Hybird | Yes | Yes | <https://journals.sagepub.com/home/caj> |
| **51** | Clinical and Translational Radiation Oncology | Elsevier | Europe | 6～12 | Open | No | Yes | <https://www.sciencedirect.com/journal/clinical-and-translational-radiation-oncology> |
| **52** | INTERNATIONAL JOURNAL OF HYPERTHERMIA | Other | Europe | 6～12 | Open | No | Yes | <https://www.tandfonline.com/journals/ihyt20?utm_source=TFO&utm_medium=cms&utm_campaign=JRA25531> |
| **53** | MOLECULAR IMAGING AND BIOLOGY | Springer | North America | 6～12 | Hybird | Yes | Yes | <https://www.springer.com/journal/11307> |
| **54** | NUCLEAR MEDICINE AND BIOLOGY | Elsevier | North America | 6～12 | Hybird | Yes | Yes | <https://www.sciencedirect.com/journal/nuclear-medicine-and-biology> |
| **55** | STRAHLENTHERAPIE UND ONKOLOGIE | Springer | Europe | ≥12 | Hybird | No | Yes | <https://www.springer.com/journal/66> |
| **56** | Ultrasonography | Society | Aisa | <6 | Open | Yes | Yes | [https://www.e-ultrasonography.org](https://www.e-ultrasonography.org/) |
| **57** | International Journal of Computer Assisted Radiology and Surgery | Springer | Europe | 6～12 | Hybird | No | No | <https://www.springer.com/journal/11548> |
| **58** | Magnetic Resonance in Medical Sciences | Society | Aisa | <6 | Open | Yes | Yes | <https://www.jstage.jst.go.jp/browse/mrms> |
| **59** | CARDIOVASCULAR AND INTERVENTIONAL RADIOLOGY | Springer | North America | ≥12 | Hybird | No | Yes | <https://www.springer.com/journal/270> |
| **60** | JOURNAL OF VASCULAR AND INTERVENTIONAL RADIOLOGY | Elsevier | North America | ≥12 | Hybird | No | Yes | <https://www.sciencedirect.com/journal/journal-of-vascular-and-interventional-radiology> |
| **61** | NMR IN BIOMEDICINE | Wiley | Europe | ≥12 | Hybird | No | No | <https://analyticalsciencejournals.onlinelibrary.wiley.com/journal/10991492/> |
| **62** | ULTRASOUND IN MEDICINE AND BIOLOGY | Elsevier | Europe | ≥12 | Hybird | No | Yes | <https://www.sciencedirect.com/journal/ultrasound-in-medicine-and-biology> |
| **63** | Clinical Neuroradiology | Springer | Europe | <6 | Hybird | No | Yes | <https://www.springer.com/journal/62> |
| **64** | Molecular Imaging | Other | North America | <6 | Open | No | Yes | <https://www.hindawi.com/journals/moi/> |
| **65** | NEURORADIOLOGY | Springer | Europe | 6～12 | Hybird | No | Yes | <https://www.springer.com/journal/234/> |
| **66** | Quantitative Imaging in Medicine and Surgery | Other | Aisa | ≥12 | Open | Yes | No | [https://qims.amegroups.org](https://qims.amegroups.org/) |
| **67** | BMC MEDICAL IMAGING | Springer | Europe | <6 | Open | Yes | No | [https://bmcmedimaging.biomedcentral.com](https://bmcmedimaging.biomedcentral.com/) |
| **68** | ANNALS OF NUCLEAR MEDICINE | Springer | Aisa | 6～12 | Hybird | Yes | Yes | <https://www.springer.com/journal/12149/> |
| **69** | BRITISH JOURNAL OF RADIOLOGY | Society | Europe | ≥12 | Hybird | Yes | Yes | <https://www.birpublications.org/journal/bjr> |
| **70** | CLINICAL RADIOLOGY | Elsevier | Europe | ≥12 | Hybird | Yes | Yes | [https://www.clinicalradiologyonline.net](https://www.clinicalradiologyonline.net/) |
| **71** | INTERNATIONAL JOURNAL OF RADIATION BIOLOGY | Other | Europe | ≥12 | Hybird | No | No | <https://www.tandfonline.com/journals/irab20> |
| **72** | Dose-Response | Other | North America | <6 | Open | No | Yes | <https://journals.sagepub.com/home/dosb> |
| **73** | Journal of Innovative Optical Health Sciences | Other | Aisa | 6～12 | Open | No | No | <https://www.worldscientific.com/worldscinet/jiohs> |
| **74** | MAGNETIC RESONANCE IMAGING | Elsevier | North America | 6～12 | Hybird | Yes | No | <https://www.sciencedirect.com/journal/magnetic-resonance-imaging> |
| **75** | Abdominal Radiology | Springer | North America | ≥12 | Hybird | Yes | Yes | <https://www.springer.com/journal/261> |
| **76** | JOURNAL OF NEUROIMAGING | Wiley | North America | 6～12 | Hybird | No | Yes | <https://onlinelibrary.wiley.com/journal/15526569> |
| **77** | JOURNAL OF NUCLEAR CARDIOLOGY | Springer | North America | 6～12 | Hybird | No | Yes | <https://www.springer.com/journal/12350> |
| **78** | Radiology and Oncology | Other | Europe | <6 | Open | No | Yes | <https://www.radioloncol.com/index.php/ro> |
| **79** | Current Radiopharmaceuticals | Other | Europe | <6 | Hybird | No | No | <https://www.eurekaselect.com/journal/95> |
| **80** | JOURNAL OF ULTRASOUND IN MEDICINE | Wiley | North America | ≥12 | Hybird | No | Yes | <https://onlinelibrary.wiley.com/journal/15509613> |
| **81** | MAGNETIC RESONANCE MATERIALS IN PHYSICS BIOLOGY AND MEDICINE | Springer | North America | 6～12 | Hybird | Yes | Yes | <https://www.springer.com/journal/10334> |
| **82** | PEDIATRIC RADIOLOGY | Springer | North America | ≥12 | Hybird | No | Yes | <https://www.springer.com/journal/247> |
| **83** | ULTRASONIC IMAGING | Other | North America | <6 | Hybird | No | No | <https://journals.sagepub.com/home/uix> |
| **84** | HEALTH PHYSICS | Lippincott Williams & Wilkins | North America | 6～12 | Hybird | No | Yes | <https://journals.lww.com/health-physics/pages/default.aspx> |
| **85** | Clinical and Translational Imaging | Springer | Europe | 6～12 | Hybird | Yes | Yes | <https://www.springer.com/journal/40336/> |
| **86** | Clinical Imaging | Elsevier | North America | 6～12 | Hybird | Yes | No | <https://www.sciencedirect.com/journal/clinical-imaging/> |
| **87** | Diagnostic and Interventional Radiology | Other | Europe | 6～12 | Open | Yes | Yes | [https://www.dirjournal.org](https://www.dirjournal.org/) |
| **88** | INTERNATIONAL JOURNAL OF CARDIOVASCULAR IMAGING | Springer | North America | 6～12 | Hybird | No | No | <https://www.springer.com/journal/10554> |
| **89** | Japanese Journal of Radiology | Springer | Aisa | 6～12 | Hybird | Yes | Yes | <https://www.springer.com/journal/11604> |
| **90** | Journal of Applied Clinical Medical Physics | Wiley | North America | 6～12 | Open | Yes | Yes | <https://aapm.onlinelibrary.wiley.com/journal/15269914/> |
| **91** | SKELETAL RADIOLOGY | Springer | North America | ≥12 | Hybird | No | Yes | <https://www.springer.com/journal/256> |
| **92** | JOURNAL OF RADIATION RESEARCH | Other | Aisa | 6～12 | Open | No | No | <https://www.sciencedirect.com/journal/journal-of-radiation-research-and-applied-sciences> |
| **93** | Journal of the Belgian Society of Radiology | Other | Europe | <6 | Open | Yes | Yes | [https://jbsr.be](https://jbsr.be/) |
| **94** | Zeitschrift fur Medizinische Physik | Elsevier | Europe | <6 | Open | Yes | Yes | <https://www.sciencedirect.com/journal/zeitschrift-fur-medizinische-physik/> |
| **95** | Brachytherapy | Elsevier | North America | <6 | Hybird | No | Yes | <https://www.sciencedirect.com/journal/brachytherapy> |
| **96** | Tomography | Other | Europe | 6～12 | Open | Yes | No | <https://www.mdpi.com/journal/tomography> |
| **97** | Journal of Medical Ultrasonics | Springer | Aisa | <6 | Hybird | Yes | Yes | <https://www.springer.com/journal/10396> |
| **98** | INTERVENTIONAL NEURORADIOLOGY | Other | Europe | <6 | Hybird | No | No | <https://journals.sagepub.com/home/ine> |
| **99** | Medical Ultrasonography | Society | Europe | <6 | Open | No | Yes | <https://www.medultrason.ro/medultrason/index.php/medultrason> |
| **100** | RADIATION AND ENVIRONMENTAL BIOPHYSICS | Springer | Europe | <6 | Hybird | No | No | <https://www.springer.com/journal/411> |
| **101** | Applied Radiation and Isotopes | Elsevier | North America | ≥12 | Hybird | No | No | <https://www.sciencedirect.com/journal/applied-radiation-and-isotopes> |
| **102** | Journal of Medical Imaging and Radiation Oncology | Wiley | Aisa | 6～12 | Hybird | Yes | Yes | <https://onlinelibrary.wiley.com/journal/17549485/> |
| **103** | Hellenic Journal of Nuclear Medicine | Society | Europe | <6 | Open | Yes | Yes | <https://www.nuclmed.gr/about/> |
| **104** | JOURNAL OF RADIOLOGICAL PROTECTION | Other | Europe | <6 | Hybird | No | Yes | <https://iopscience.iop.org/journal/0952-4746> |
| **105** | NUCLEAR MEDICINE COMMUNICATIONS | Lippincott Williams & Wilkins | North America | ≥12 | Hybird | Yes | Yes | <https://journals.lww.com/nuclearmedicinecomm/Pages/default.aspx> |
| **106** | QUARTERLY JOURNAL OF NUCLEAR MEDICINE AND MOLECULAR IMAGING | Other | Europe | <6 | Hybird | Yes | Yes | <https://www.minervamedica.it/en/journals/nuclear-med-molecular-imaging/index.php> |
| **107** | Current Medical Imaging | Other | Aisa | 6～12 | Open | Yes | No | <https://www.eurekaselect.com/journal/33> |
| **108** | Journal of Contemporary Brachytherapy | Other | Europe | <6 | Open | No | Yes | <https://www.termedia.pl/Journal/-54> |
| **109** | SURGICAL AND RADIOLOGIC ANATOMY | Springer | Europe | 6～12 | Hybird | No | No | <https://www.springer.com/journal/276> |
| **110** | ACTA RADIOLOGICA | Other | Europe | ≥12 | Hybird | Yes | Yes | <https://journals.sagepub.com/home/acr> |
| **111** | JOURNAL OF COMPUTER ASSISTED TOMOGRAPHY | Lippincott Williams & Wilkins | North America | 6～12 | Hybird | Yes | No | <https://journals.lww.com/jcat/pages/default.aspx> |
| **112** | Ultrasound Quarterly | Lippincott Williams & Wilkins | North America | <6 | Hybird | Yes | Yes | <https://journals.lww.com/ultrasound-quarterly/pages/default.aspx> |
| **113** | Medical Dosimetry | Elsevier | North America | <6 | Hybird | No | Yes | <https://www.sciencedirect.com/journal/medical-dosimetry> |
| **114** | RADIATION PROTECTION DOSIMETRY | Other | Europe | ≥12 | Hybird | No | No | <https://academic.oup.com/rpd> |
| **115** | JOURNAL OF CLINICAL ULTRASOUND | Wiley | North America | 6～12 | Hybird | No | No | <https://onlinelibrary.wiley.com/journal/10970096> |
| **116** | CONCEPTS IN MAGNETIC RESONANCE PART A | Other | North America | <6 | Hybird | No | No | <https://www.hindawi.com/journals/cmra/> |
| **117** | International Journal of Radiation Research | Other | Aisa | <6 | Open | Yes | No | <http://ijrr.com/page.php?slct_pg_id=12&sid=1&slc_lang=en> |

**Supplementary Table S3 Five endorsement level defined with examples in radiological journals**

| **Endorsement level** | **Definition** | **Example** | **Journal** | **Source** |
| --- | --- | --- | --- | --- |
| **Active strong** | A requirement of a completed checklist and/or a flow diagram with article submission (e.g., “must”, “should be uploaded”). | E. g. 1: Your paper will be sent back if this checklist is not included upon first submission (STARD, CONSORT, PRISMA, STROBE).  E. g. 2: For biomedical and biological research, the checklist below must be completed before peer review, and made available to the Editors and reviewers: Randomized controlled trials (CONSORT) | E. g. 1: *Radiology*  E. g. 2:  *Journal of Cardiovascular Magnetic Resonance* | E. g. 1: <https://pubs.rsna.org/page/radiology/author-instructions>  E. g. 2:  <https://jcmr-online.biomedcentral.com/submission-guidelines> |
| **Active weak** | A suggestion that authors are “encouraged” or “should” reference or follow a specific guideline; Priority publication if follow a specific guideline. | E. g. 1: Authors are recommended to adhere to the minimum reporting guidelines hosted by the EQUATOR Network when preparing their manuscript (CONSOTR, SPIRIT, STROBE, etc.)  E. g. 2: According to the type of the study, you are advised to consult the following abstract checklists: STARD, STROBE, PRISMA-DTA, PRISMA, TRIPOD, CONSORT). (“Passive moderate”) Adherence to applicable reporting guidelines (see EQUATOR Network (“Passive weak”) or CLAIM for artificial intelligence papers (“Active weak”)) is strongly recommended. | E. g. 1: *European Journal of Nuclear Medicine and Molecular Imaging*  E. g. 2: *European Radiology* | E. g. 1: <https://www.springer.com/journal/259/submission-guidelines>  E. g. 2: <https://www.european-radiology.org/content-er/uploads/2022/12/European-Radiology_Manuscript-requirements_Edition-1.pdf> |
| **Passive moderate** | A suggestion that authors should adhere to “relevant” reporting guidelines; Abstracts are required to follow a specific guideline. | E. g: According to the type of the study, you are advised to consult the following abstract checklists: STARD, STROBE, PRISMA-DTA, PRISMA, TRIPOD, CONSORT). (“Passive moderate”) Adherence to applicable reporting guidelines (see EQUATOR Network (“Passive weak”) or CLAIM for artificial intelligence papers (“Active weak”)) is strongly recommended. | E. g: *European Radiology* | E. g: <https://www.european-radiology.org/content-er/uploads/2022/12/European-Radiology_Manuscript-requirements_Edition-1.pdf> |
| **Passive weak** | References documents (e.g., ICMJE, or EQUATOR Network) which mention reporting guidelines. If the instructions for authors mentions the ICMJE (<https://www.icmje.org>), the 4 general reporting guidelines mentioned by ICMJE (CONSORT, STROBE, PRISMA, and STARD) will be rated as “Passive weak”. However, the mention of ICMJE in a specific section (such as authorship, conflict of interest, ethics, etc.), were not considered as appropriate. If the instructions for authors mentions the EQUATOR Network (<https://www.equator-network.org>), the 15 general reporting guidelines for main study types on the homepage of EQUATOR Network will be rated as “Passive weak”. | E. g. 1: Studies submitted to *Korean Journal of Radiology* should also follow established guidelines for reporting research studies (<http://www.equator-network.org>).  E. g. 2: According to the type of the study, you are advised to consult the following abstract checklists: STARD, STROBE, PRISMA-DTA, PRISMA, TRIPOD, CONSORT). (“Passive moderate”) Adherence to applicable reporting guidelines (see EQUATOR Network (“Passive weak”) or CLAIM for artificial intelligence papers (“Active weak”)) is strongly recommended. | E. g. 1: *Korean Journal of Radiology*  E. g. 2: *European Radiology* | E. g. 1: <https://www.kjronline.org/index.php?body=Instruction>  E. g. 2: <https://www.european-radiology.org/content-er/uploads/2022/12/European-Radiology_Manuscript-requirements_Edition-1.pdf> |
| **None** | No mention of any reporting guidelines. | N. a. | N. a. | N. a. |

**Supplementary Table S4 Endorsement level rating of reporting guidelines of included journals**

| **No** | **Journal** | **CONSORT** | **STROBE** | **PRISMA** | **SPIRIT** | **PRISMA-P** | **STARD** | **TRIPOD** | **CARE** | **ARRIVE** | **AGREE** | **RIGHT** | **SRQR** | **COREQ** | **SQUIRE** | **CHEERS** | **CONSORT-AI** | **SPIRIT-AI** | **FUTURE-AI** | **MI-CLAIM** | **MINIMAR** | **CLAIM** | **MAIC-10** | **RQS** | **IBSI** | **CLEAR** |
| --- | --- | --- | --- | --- | --- | --- | --- | --- | --- | --- | --- | --- | --- | --- | --- | --- | --- | --- | --- | --- | --- | --- | --- | --- | --- | --- |
| **1** | RADIOLOGY | 1 | 1 | 1 | 0 | 0 | 1 | 0 | 0 | 0 | 0 | 0 | 0 | 0 | 0 | 0 | 0 | 0 | 0 | 0 | 0 | 0 | 0 | 0 | 0 | 0 |
| **2** | JACC-CARDIOVASC IMAG | 0 | 0 | 0 | 0 | 0 | 0 | 0 | 0 | 0 | 0 | 0 | 0 | 0 | 0 | 0 | 0 | 0 | 0 | 0 | 0 | 0 | 0 | 0 | 0 | 0 |
| **3** | MED IMAGE ANAL | 0 | 0 | 0 | 0 | 0 | 0 | 0 | 0 | 0 | 0 | 0 | 0 | 0 | 0 | 0 | 0 | 0 | 0 | 0 | 0 | 0 | 0 | 0 | 0 | 0 |
| **4** | CLIN NUCL MED | 0 | 0 | 0 | 0 | 0 | 0 | 0 | 0 | 0 | 0 | 0 | 0 | 0 | 0 | 0 | 0 | 0 | 0 | 0 | 0 | 0 | 0 | 0 | 0 | 0 |
| **5** | IEEE T MED IMAGING | 0 | 0 | 0 | 0 | 0 | 0 | 0 | 0 | 0 | 0 | 0 | 0 | 0 | 0 | 0 | 0 | 0 | 0 | 0 | 0 | 0 | 0 | 0 | 0 | 0 |
| **6** | J NUCL MED | 1 | 4 | 1 | 0 | 0 | 1 | 0 | 0 | 0 | 0 | 0 | 0 | 0 | 0 | 0 | 0 | 0 | 0 | 0 | 0 | 0 | 0 | 0 | 0 | 0 |
| **7** | EUR J NUCL MED MOL I | 2 | 2 | 2 | 2 | 2 | 2 | 2 | 2 | 2 | 2 | 2 | 2 | 2 | 2 | 2 | 0 | 0 | 0 | 0 | 0 | 0 | 0 | 0 | 0 | 0 |
| **8** | RADIOL MED | 2 | 2 | 2 | 2 | 2 | 2 | 2 | 2 | 2 | 2 | 2 | 2 | 2 | 2 | 2 | 0 | 0 | 0 | 0 | 0 | 0 | 0 | 0 | 0 | 0 |
| **9** | PHOTOACOUSTICS | 1 | 0 | 0 | 0 | 0 | 0 | 0 | 0 | 0 | 0 | 0 | 0 | 0 | 0 | 0 | 0 | 0 | 0 | 0 | 0 | 0 | 0 | 0 | 0 | 0 |
| **10** | CIRC-CARDIOVASC IMAG | 2 | 2 | 2 | 0 | 0 | 2 | 2 | 0 | 0 | 0 | 0 | 0 | 0 | 0 | 0 | 0 | 0 | 0 | 0 | 0 | 0 | 0 | 0 | 0 | 0 |
| **11** | ULTRASOUND OBST GYN | 1 | 1 | 1 | 0 | 0 | 1 | 1 | 0 | 0 | 0 | 0 | 0 | 0 | 0 | 1 | 0 | 0 | 0 | 0 | 0 | 0 | 0 | 0 | 0 | 0 |
| **12** | INT J RADIAT ONCOL | 2 | 2 | 2 | 0 | 0 | 0 | 0 | 0 | 0 | 0 | 0 | 0 | 0 | 0 | 0 | 0 | 0 | 0 | 0 | 0 | 0 | 0 | 0 | 0 | 0 |
| **13** | INVEST RADIOL | 0 | 0 | 0 | 0 | 0 | 0 | 0 | 0 | 0 | 0 | 0 | 0 | 0 | 0 | 0 | 0 | 0 | 0 | 0 | 0 | 0 | 0 | 0 | 0 | 0 |
| **14** | J CARDIOVASC MAGN R | 1 | 2 | 2 | 2 | 2 | 2 | 2 | 2 | 2 | 4 | 4 | 4 | 2 | 4 | 2 | 0 | 0 | 0 | 0 | 0 | 0 | 0 | 0 | 0 | 0 |
| **15** | EUR HEART J-CARD IMG | 2 | 2 | 2 | 2 | 4 | 2 | 4 | 4 | 2 | 4 | 4 | 2 | 4 | 2 | 2 | 0 | 0 | 0 | 0 | 0 | 0 | 0 | 0 | 0 | 0 |
| **16** | EUR RADIOL | 3 | 3 | 3 | 4 | 4 | 3 | 3 | 4 | 4 | 4 | 4 | 4 | 4 | 4 | 4 | 4 | 0 | 0 | 0 | 0 | 2 | 0 | 0 | 0 | 0 |
| **17** | COMPUT MED IMAG GRAP | 0 | 0 | 0 | 0 | 0 | 0 | 0 | 0 | 0 | 0 | 0 | 0 | 0 | 0 | 0 | 0 | 0 | 0 | 0 | 0 | 0 | 0 | 0 | 0 | 0 |
| **18** | NEUROIMAGE | 0 | 0 | 0 | 0 | 0 | 0 | 0 | 0 | 0 | 0 | 0 | 0 | 0 | 0 | 0 | 0 | 0 | 0 | 0 | 0 | 0 | 0 | 0 | 0 | 0 |
| **19** | RADIOTHER ONCOL | 1 | 4 | 4 | 0 | 0 | 4 | 0 | 0 | 0 | 0 | 0 | 0 | 0 | 0 | 0 | 0 | 0 | 0 | 0 | 0 | 0 | 0 | 0 | 0 | 0 |
| **20** | DIAGN INTERV IMAG | 1 | 0 | 0 | 0 | 0 | 0 | 0 | 0 | 0 | 0 | 0 | 0 | 0 | 0 | 0 | 0 | 0 | 0 | 0 | 0 | 0 | 0 | 0 | 0 | 0 |
| **21** | J CARDIOVASC COMPUT | 0 | 0 | 0 | 0 | 0 | 0 | 0 | 0 | 0 | 0 | 0 | 0 | 0 | 0 | 0 | 0 | 0 | 0 | 0 | 0 | 0 | 0 | 0 | 0 | 0 |
| **22** | AM J ROENTGENOL | 0 | 0 | 2 | 0 | 0 | 0 | 0 | 0 | 0 | 0 | 0 | 0 | 0 | 0 | 0 | 0 | 0 | 0 | 0 | 0 | 0 | 0 | 0 | 0 | 0 |
| **23** | CANCER IMAGING | 1 | 2 | 2 | 2 | 2 | 2 | 2 | 2 | 2 | 4 | 4 | 4 | 2 | 4 | 2 | 0 | 0 | 0 | 0 | 0 | 0 | 0 | 0 | 0 | 0 |
| **24** | ACAD RADIOL | 1 | 0 | 0 | 0 | 0 | 0 | 0 | 0 | 0 | 0 | 0 | 0 | 0 | 0 | 0 | 0 | 0 | 0 | 0 | 0 | 0 | 0 | 0 | 0 | 0 |
| **25** | HUM BRAIN MAPP | 0 | 0 | 0 | 0 | 0 | 0 | 0 | 0 | 0 | 0 | 0 | 0 | 0 | 0 | 0 | 0 | 0 | 0 | 0 | 0 | 0 | 0 | 0 | 0 | 0 |
| **26** | KOREAN J RADIOL | 4 | 4 | 4 | 4 | 4 | 4 | 4 | 4 | 4 | 4 | 4 | 4 | 4 | 4 | 4 | 0 | 0 | 0 | 0 | 0 | 0 | 0 | 0 | 0 | 0 |
| **27** | INSIGHTS IMAGING | 3 | 3 | 3 | 4 | 4 | 3 | 3 | 4 | 4 | 4 | 4 | 4 | 4 | 4 | 4 | 4 | 0 | 0 | 0 | 0 | 0 | 0 | 0 | 0 | 0 |
| **28** | J AM COLL RADIOL | 2 | 2 | 2 | 0 | 0 | 0 | 0 | 0 | 0 | 0 | 0 | 0 | 0 | 2 | 0 | 0 | 0 | 0 | 0 | 0 | 2 | 0 | 0 | 0 | 0 |
| **29** | J DIGIT IMAGING | 2 | 2 | 2 | 2 | 2 | 2 | 2 | 2 | 2 | 2 | 2 | 2 | 2 | 2 | 2 | 0 | 0 | 0 | 0 | 0 | 0 | 0 | 0 | 0 | 0 |
| **30** | J MAGN RESON IMAGING | 2 | 0 | 2 | 0 | 0 | 2 | 0 | 0 | 0 | 0 | 0 | 0 | 0 | 0 | 0 | 0 | 0 | 0 | 0 | 0 | 0 | 0 | 0 | 0 | 0 |
| **31** | PHYS ENG SCI MED | 2 | 2 | 2 | 2 | 2 | 2 | 2 | 2 | 2 | 2 | 2 | 2 | 2 | 2 | 2 | 0 | 0 | 0 | 0 | 0 | 0 | 0 | 0 | 0 | 0 |
| **32** | ULTRASONICS | 0 | 0 | 0 | 0 | 0 | 0 | 0 | 0 | 0 | 0 | 0 | 0 | 0 | 0 | 0 | 0 | 0 | 0 | 0 | 0 | 0 | 0 | 0 | 0 | 0 |
| **33** | EJNMMI PHYS | 2 | 2 | 2 | 4 | 4 | 2 | 4 | 4 | 2 | 4 | 4 | 4 | 4 | 4 | 4 | 0 | 0 | 0 | 0 | 0 | 0 | 0 | 0 | 0 | 0 |
| **34** | MED PHYS | 0 | 0 | 0 | 0 | 0 | 0 | 0 | 0 | 2 | 0 | 0 | 0 | 0 | 0 | 0 | 0 | 0 | 0 | 0 | 0 | 0 | 0 | 0 | 0 | 0 |
| **35** | RADIAT ONCOL | 1 | 2 | 2 | 2 | 2 | 2 | 2 | 2 | 2 | 4 | 4 | 4 | 2 | 4 | 2 | 0 | 0 | 0 | 0 | 0 | 0 | 0 | 0 | 0 | 0 |
| **36** | AM J NEURORADIOL | 0 | 0 | 2 | 0 | 0 | 2 | 0 | 0 | 0 | 0 | 0 | 0 | 0 | 0 | 0 | 0 | 0 | 0 | 0 | 0 | 0 | 0 | 0 | 0 | 0 |
| **37** | J BIOMED OPT | 0 | 0 | 0 | 0 | 0 | 0 | 0 | 0 | 0 | 0 | 0 | 0 | 0 | 0 | 0 | 0 | 0 | 0 | 0 | 0 | 0 | 0 | 0 | 0 | 0 |
| **38** | J NEURORADIOLOGY | 0 | 0 | 0 | 0 | 0 | 0 | 0 | 0 | 0 | 0 | 0 | 0 | 0 | 0 | 0 | 0 | 0 | 0 | 0 | 0 | 0 | 0 | 0 | 0 | 0 |
| **39** | PHYS MED BIOL | 0 | 0 | 0 | 0 | 0 | 0 | 0 | 0 | 0 | 0 | 0 | 0 | 0 | 0 | 0 | 0 | 0 | 0 | 0 | 0 | 0 | 0 | 0 | 0 | 0 |
| **40** | BIOMED OPT EXPRESS | 0 | 0 | 0 | 0 | 0 | 0 | 0 | 0 | 0 | 0 | 0 | 0 | 0 | 0 | 0 | 0 | 0 | 0 | 0 | 0 | 0 | 0 | 0 | 0 | 0 |
| **41** | CANCER BIOTHER RADIO | 4 | 4 | 4 | 4 | 4 | 4 | 4 | 4 | 4 | 4 | 4 | 4 | 4 | 4 | 4 | 0 | 0 | 0 | 0 | 0 | 0 | 0 | 0 | 0 | 0 |
| **42** | PHYS MEDICA | 1 | 0 | 0 | 0 | 0 | 0 | 0 | 0 | 0 | 0 | 0 | 0 | 0 | 0 | 0 | 0 | 0 | 0 | 0 | 0 | 0 | 0 | 0 | 0 | 0 |
| **43** | RADIAT RES | 0 | 0 | 0 | 0 | 0 | 0 | 0 | 0 | 0 | 0 | 0 | 0 | 0 | 0 | 0 | 0 | 0 | 0 | 0 | 0 | 0 | 0 | 0 | 0 | 0 |
| **44** | DENTOMAXILLOFAC RAD | 1 | 1 | 1 | 2 | 4 | 1 | 4 | 2 | 2 | 2 | 4 | 2 | 4 | 2 | 2 | 0 | 0 | 0 | 0 | 0 | 0 | 0 | 0 | 0 | 0 |
| **45** | EUR J RADIOL | 0 | 0 | 0 | 0 | 0 | 0 | 0 | 0 | 0 | 0 | 0 | 0 | 0 | 0 | 0 | 0 | 0 | 0 | 0 | 0 | 0 | 0 | 0 | 0 | 0 |
| **46** | J THORAC IMAG | 0 | 0 | 0 | 0 | 0 | 0 | 0 | 0 | 0 | 0 | 0 | 0 | 0 | 0 | 0 | 0 | 0 | 0 | 0 | 0 | 0 | 0 | 0 | 0 | 0 |
| **47** | MAGN RESON MED | 0 | 0 | 0 | 0 | 0 | 0 | 0 | 0 | 0 | 0 | 0 | 0 | 0 | 0 | 0 | 0 | 0 | 0 | 0 | 0 | 0 | 0 | 0 | 0 | 0 |
| **48** | PRACT RADIAT ONCOL | 2 | 2 | 2 | 0 | 0 | 0 | 0 | 0 | 0 | 0 | 0 | 0 | 0 | 0 | 0 | 0 | 0 | 0 | 0 | 0 | 0 | 0 | 0 | 0 | 0 |
| **49** | EJNMMI RES | 2 | 2 | 2 | 4 | 4 | 2 | 4 | 4 | 2 | 4 | 4 | 4 | 4 | 4 | 4 | 0 | 0 | 0 | 0 | 0 | 0 | 0 | 0 | 0 | 0 |
| **50** | CAN ASSOC RADIOL J | 2 | 4 | 2 | 4 | 4 | 2 | 4 | 4 | 4 | 4 | 4 | 4 | 4 | 4 | 4 | 0 | 0 | 0 | 0 | 0 | 0 | 0 | 0 | 0 | 0 |
| **51** | CLIN TRANSL RAD ONCO | 1 | 4 | 4 | 0 | 0 | 4 | 0 | 0 | 0 | 0 | 0 | 0 | 0 | 0 | 0 | 0 | 0 | 0 | 0 | 0 | 0 | 0 | 0 | 0 | 0 |
| **52** | INT J HYPERTHER | 4 | 4 | 4 | 4 | 4 | 4 | 4 | 4 | 4 | 4 | 4 | 4 | 4 | 4 | 4 | 0 | 0 | 0 | 0 | 0 | 0 | 0 | 0 | 0 | 0 |
| **53** | MOL IMAGING BIOL | 0 | 0 | 0 | 0 | 0 | 0 | 0 | 0 | 0 | 0 | 0 | 0 | 0 | 0 | 0 | 0 | 0 | 0 | 0 | 0 | 0 | 0 | 0 | 0 | 0 |
| **54** | NUCL MED BIOL | 1 | 0 | 0 | 0 | 0 | 0 | 0 | 0 | 0 | 0 | 0 | 0 | 0 | 0 | 0 | 0 | 0 | 0 | 0 | 0 | 0 | 0 | 0 | 0 | 0 |
| **55** | STRAHLENTHER ONKOL | 2 | 2 | 2 | 2 | 2 | 2 | 2 | 2 | 2 | 2 | 2 | 2 | 2 | 2 | 2 | 0 | 0 | 0 | 0 | 0 | 0 | 0 | 0 | 0 | 0 |
| **56** | ULTRASONOGRAPHY | 2 | 2 | 2 | 0 | 0 | 2 | 0 | 0 | 0 | 0 | 0 | 0 | 0 | 0 | 0 | 0 | 0 | 0 | 0 | 0 | 0 | 0 | 0 | 0 | 0 |
| **57** | INT J COMPUT ASS RAD | 2 | 2 | 2 | 2 | 2 | 2 | 2 | 2 | 2 | 2 | 2 | 2 | 2 | 2 | 2 | 0 | 0 | 0 | 0 | 0 | 0 | 0 | 0 | 0 | 0 |
| **58** | MAGN RESON MED SCI | 0 | 0 | 0 | 0 | 0 | 0 | 0 | 0 | 0 | 0 | 0 | 0 | 0 | 0 | 0 | 0 | 0 | 0 | 0 | 0 | 0 | 0 | 0 | 0 | 0 |
| **59** | CARDIOVASC INTER RAD | 2 | 2 | 2 | 2 | 2 | 2 | 2 | 2 | 2 | 2 | 2 | 2 | 2 | 2 | 2 | 0 | 0 | 0 | 0 | 0 | 0 | 0 | 0 | 0 | 0 |
| **60** | J VASC INTERV RADIOL | 1 | 2 | 1 | 0 | 0 | 0 | 0 | 0 | 1 | 0 | 0 | 0 | 0 | 0 | 0 | 0 | 0 | 0 | 0 | 0 | 0 | 0 | 0 | 0 | 0 |
| **61** | NMR BIOMED | 0 | 0 | 0 | 0 | 0 | 0 | 0 | 0 | 0 | 0 | 0 | 0 | 0 | 0 | 0 | 0 | 0 | 0 | 0 | 0 | 0 | 0 | 0 | 0 | 0 |
| **62** | ULTRASOUND MED BIOL | 2 | 0 | 2 | 0 | 0 | 2 | 0 | 0 | 0 | 0 | 0 | 0 | 0 | 0 | 0 | 0 | 0 | 0 | 0 | 0 | 0 | 0 | 0 | 0 | 0 |
| **63** | CLIN NEURORADIOL | 2 | 2 | 2 | 2 | 2 | 2 | 2 | 2 | 2 | 2 | 2 | 2 | 2 | 2 | 2 | 0 | 0 | 0 | 0 | 0 | 0 | 0 | 0 | 0 | 0 |
| **64** | MOL IMAGING | 1 | 1 | 1 | 0 | 0 | 1 | 0 | 1 | 1 | 0 | 0 | 1 | 0 | 0 | 0 | 0 | 0 | 0 | 0 | 0 | 0 | 0 | 0 | 0 | 0 |
| **65** | NEURORADIOLOGY | 2 | 2 | 2 | 4 | 4 | 2 | 4 | 4 | 4 | 4 | 4 | 4 | 4 | 4 | 4 | 0 | 0 | 0 | 0 | 0 | 0 | 0 | 0 | 0 | 0 |
| **66** | QUANT IMAG MED SURG | 1 | 1 | 1 | 4 | 4 | 1 | 1 | 4 | 1 | 4 | 1 | 4 | 4 | 4 | 4 | 0 | 0 | 0 | 0 | 0 | 0 | 0 | 0 | 0 | 0 |
| **67** | BMC MED IMAGING | 1 | 2 | 2 | 2 | 2 | 2 | 2 | 2 | 2 | 4 | 4 | 4 | 2 | 4 | 2 | 0 | 0 | 0 | 0 | 0 | 0 | 0 | 0 | 0 | 0 |
| **68** | ANN NUCL MED | 4 | 4 | 4 | 0 | 0 | 4 | 0 | 0 | 0 | 0 | 0 | 0 | 0 | 0 | 0 | 0 | 0 | 0 | 0 | 0 | 0 | 0 | 0 | 0 | 0 |
| **69** | BRIT J RADIOL | 1 | 1 | 1 | 2 | 4 | 1 | 4 | 2 | 2 | 2 | 4 | 2 | 4 | 2 | 2 | 0 | 0 | 0 | 0 | 0 | 0 | 0 | 0 | 0 | 0 |
| **70** | CLIN RADIOL | 0 | 0 | 0 | 0 | 0 | 0 | 0 | 0 | 0 | 0 | 0 | 0 | 0 | 0 | 0 | 0 | 0 | 0 | 0 | 0 | 0 | 0 | 0 | 0 | 0 |
| **71** | INT J RADIAT BIOL | 4 | 4 | 4 | 0 | 0 | 4 | 0 | 0 | 0 | 0 | 0 | 0 | 0 | 0 | 0 | 0 | 0 | 0 | 0 | 0 | 0 | 0 | 0 | 0 | 0 |
| **72** | DOSE-RESPONSE | 1 | 4 | 1 | 4 | 4 | 4 | 4 | 4 | 4 | 4 | 4 | 4 | 4 | 4 | 4 | 0 | 0 | 0 | 0 | 0 | 0 | 0 | 0 | 0 | 0 |
| **73** | J INNOV OPT HEAL SCI | 0 | 0 | 0 | 0 | 0 | 0 | 0 | 0 | 0 | 0 | 0 | 0 | 0 | 0 | 0 | 0 | 0 | 0 | 0 | 0 | 0 | 0 | 0 | 0 | 0 |
| **74** | MAGN RESON IMAGING | 0 | 0 | 0 | 0 | 0 | 0 | 0 | 0 | 0 | 0 | 0 | 0 | 0 | 0 | 0 | 0 | 0 | 0 | 0 | 0 | 0 | 0 | 0 | 0 | 0 |
| **75** | ABDOM RADIOL | 2 | 2 | 2 | 2 | 2 | 2 | 2 | 2 | 2 | 2 | 2 | 2 | 2 | 2 | 2 | 0 | 0 | 0 | 0 | 0 | 0 | 0 | 0 | 0 | 0 |
| **76** | J NEUROIMAGING | 0 | 0 | 0 | 0 | 0 | 0 | 0 | 0 | 0 | 0 | 0 | 0 | 0 | 0 | 0 | 0 | 0 | 0 | 0 | 0 | 0 | 0 | 0 | 0 | 0 |
| **77** | J NUCL CARDIOL | 0 | 0 | 0 | 0 | 0 | 1 | 0 | 0 | 0 | 0 | 0 | 0 | 0 | 0 | 0 | 0 | 0 | 0 | 0 | 0 | 0 | 0 | 0 | 0 | 0 |
| **78** | RADIOL ONCOL | 0 | 0 | 0 | 0 | 0 | 0 | 0 | 0 | 0 | 0 | 0 | 0 | 0 | 0 | 0 | 0 | 0 | 0 | 0 | 0 | 0 | 0 | 0 | 0 | 0 |
| **79** | CURR RADIOPHARM | 1 | 2 | 2 | 4 | 4 | 2 | 2 | 1 | 4 | 4 | 4 | 4 | 2 | 4 | 2 | 0 | 0 | 0 | 0 | 0 | 0 | 0 | 0 | 0 | 0 |
| **80** | J ULTRAS MED | 0 | 0 | 0 | 0 | 0 | 0 | 0 | 0 | 0 | 0 | 0 | 0 | 0 | 0 | 0 | 0 | 0 | 0 | 0 | 0 | 0 | 0 | 0 | 0 | 0 |
| **81** | MAGN RESON MATER PHY | 2 | 2 | 2 | 2 | 2 | 2 | 2 | 2 | 2 | 2 | 2 | 2 | 2 | 2 | 2 | 0 | 0 | 0 | 0 | 0 | 0 | 0 | 0 | 0 | 0 |
| **82** | PEDIATR RADIOL | 2 | 2 | 2 | 2 | 2 | 2 | 2 | 2 | 2 | 2 | 2 | 2 | 2 | 2 | 2 | 0 | 0 | 0 | 0 | 0 | 0 | 0 | 0 | 0 | 0 |
| **83** | ULTRASONIC IMAGING | 1 | 4 | 1 | 4 | 4 | 4 | 4 | 4 | 4 | 4 | 4 | 4 | 4 | 4 | 4 | 0 | 0 | 0 | 0 | 0 | 0 | 0 | 0 | 0 | 0 |
| **84** | HEALTH PHYS | 0 | 0 | 0 | 0 | 0 | 0 | 0 | 0 | 0 | 0 | 0 | 0 | 0 | 0 | 0 | 0 | 0 | 0 | 0 | 0 | 0 | 0 | 0 | 0 | 0 |
| **85** | CLIN TRANSL IMAGING | 2 | 2 | 2 | 2 | 2 | 2 | 2 | 2 | 2 | 2 | 2 | 2 | 2 | 2 | 2 | 0 | 0 | 0 | 0 | 0 | 0 | 0 | 0 | 0 | 0 |
| **86** | CLIN IMAG | 2 | 2 | 2 | 0 | 0 | 0 | 0 | 0 | 0 | 0 | 0 | 0 | 0 | 2 | 0 | 0 | 0 | 0 | 0 | 0 | 0 | 0 | 0 | 0 | 0 |
| **87** | DIAGN INTERV RADIOL | 2 | 2 | 2 | 0 | 0 | 2 | 0 | 0 | 0 | 0 | 0 | 0 | 0 | 0 | 0 | 0 | 0 | 0 | 0 | 0 | 0 | 0 | 0 | 0 | 0 |
| **88** | INT J CARDIOVAS IMAG | 2 | 2 | 2 | 2 | 2 | 2 | 2 | 2 | 2 | 2 | 2 | 2 | 2 | 2 | 2 | 0 | 0 | 0 | 0 | 0 | 0 | 0 | 0 | 0 | 0 |
| **89** | JPN J RADIOL | 0 | 0 | 0 | 0 | 0 | 0 | 0 | 0 | 0 | 0 | 0 | 0 | 0 | 0 | 0 | 0 | 0 | 0 | 0 | 0 | 0 | 0 | 0 | 0 | 0 |
| **90** | J APPL CLIN MED PHYS | 0 | 0 | 0 | 0 | 0 | 0 | 0 | 0 | 0 | 0 | 0 | 0 | 0 | 0 | 0 | 0 | 0 | 0 | 0 | 0 | 0 | 0 | 0 | 0 | 0 |
| **91** | SKELETAL RADIOL | 4 | 4 | 4 | 0 | 0 | 4 | 0 | 0 | 0 | 0 | 0 | 0 | 0 | 0 | 0 | 0 | 0 | 0 | 0 | 0 | 0 | 0 | 0 | 0 | 0 |
| **92** | J RADIAT RES | 0 | 0 | 0 | 0 | 0 | 0 | 0 | 0 | 0 | 0 | 0 | 0 | 0 | 0 | 0 | 0 | 0 | 0 | 0 | 0 | 0 | 0 | 0 | 0 | 0 |
| **93** | J BELG SOC RADIOL | 4 | 4 | 4 | 0 | 0 | 4 | 0 | 0 | 0 | 0 | 0 | 0 | 0 | 0 | 0 | 0 | 0 | 0 | 0 | 0 | 0 | 0 | 0 | 0 | 0 |
| **94** | Z MED PHYS | 0 | 0 | 0 | 0 | 0 | 0 | 0 | 0 | 0 | 0 | 0 | 0 | 0 | 0 | 0 | 0 | 0 | 0 | 0 | 0 | 0 | 0 | 0 | 0 | 0 |
| **95** | BRACHYTHERAPY | 0 | 0 | 0 | 0 | 0 | 0 | 0 | 0 | 0 | 0 | 0 | 0 | 0 | 0 | 0 | 0 | 0 | 0 | 0 | 0 | 0 | 0 | 0 | 0 | 0 |
| **96** | TOMOGRAPHY | 1 | 0 | 0 | 0 | 0 | 0 | 0 | 0 | 0 | 0 | 0 | 0 | 0 | 0 | 0 | 0 | 0 | 0 | 0 | 0 | 0 | 0 | 0 | 0 | 0 |
| **97** | J MED ULTRASON | 0 | 0 | 0 | 0 | 0 | 0 | 0 | 0 | 0 | 0 | 0 | 0 | 0 | 0 | 0 | 0 | 0 | 0 | 0 | 0 | 0 | 0 | 0 | 0 | 0 |
| **98** | INTERV NEURORADIOL | 1 | 4 | 1 | 4 | 4 | 4 | 4 | 4 | 4 | 4 | 4 | 4 | 4 | 4 | 4 | 0 | 0 | 0 | 0 | 0 | 0 | 0 | 0 | 0 | 0 |
| **99** | MED ULTRASON | 0 | 0 | 0 | 0 | 0 | 0 | 0 | 0 | 0 | 0 | 0 | 0 | 0 | 0 | 0 | 0 | 0 | 0 | 0 | 0 | 0 | 0 | 0 | 0 | 0 |
| **100** | RADIAT ENVIRON BIOPH | 2 | 2 | 2 | 2 | 2 | 2 | 2 | 2 | 2 | 2 | 2 | 2 | 2 | 2 | 2 | 0 | 0 | 0 | 0 | 0 | 0 | 0 | 0 | 0 | 0 |
| **101** | APPL RADIAT ISOTOPES | 0 | 0 | 0 | 0 | 0 | 0 | 0 | 0 | 0 | 0 | 0 | 0 | 0 | 0 | 0 | 0 | 0 | 0 | 0 | 0 | 0 | 0 | 0 | 0 | 0 |
| **102** | J MED IMAG RADIAT ON | 2 | 2 | 1 | 0 | 0 | 2 | 0 | 0 | 0 | 0 | 0 | 0 | 0 | 0 | 0 | 0 | 0 | 0 | 0 | 0 | 0 | 0 | 0 | 0 | 0 |
| **103** | HELL J NUCL MED | 0 | 0 | 0 | 0 | 0 | 0 | 0 | 0 | 0 | 0 | 0 | 0 | 0 | 0 | 0 | 0 | 0 | 0 | 0 | 0 | 0 | 0 | 0 | 0 | 0 |
| **104** | J RADIOL PROT | 0 | 0 | 0 | 0 | 0 | 0 | 0 | 0 | 0 | 0 | 0 | 0 | 0 | 0 | 0 | 0 | 0 | 0 | 0 | 0 | 0 | 0 | 0 | 0 | 0 |
| **105** | NUCL MED COMMUN | 0 | 0 | 0 | 0 | 0 | 0 | 0 | 0 | 0 | 0 | 0 | 0 | 0 | 0 | 0 | 0 | 0 | 0 | 0 | 0 | 0 | 0 | 0 | 0 | 0 |
| **106** | Q J NUCL MED MOL IM | 2 | 4 | 2 | 0 | 0 | 4 | 0 | 0 | 0 | 0 | 0 | 0 | 0 | 0 | 0 | 0 | 0 | 0 | 0 | 0 | 0 | 0 | 0 | 0 | 0 |
| **107** | CURR MED IMAGING | 1 | 2 | 2 | 4 | 4 | 2 | 2 | 2 | 2 | 4 | 4 | 4 | 2 | 4 | 2 | 0 | 0 | 0 | 0 | 0 | 0 | 0 | 0 | 0 | 0 |
| **108** | J CONTEMP BRACHYTHER | 4 | 4 | 4 | 0 | 0 | 4 | 0 | 0 | 0 | 0 | 0 | 0 | 0 | 0 | 0 | 0 | 0 | 0 | 0 | 0 | 0 | 0 | 0 | 0 | 0 |
| **109** | SURG RADIOL ANAT | 2 | 2 | 2 | 2 | 2 | 2 | 2 | 2 | 2 | 2 | 2 | 2 | 2 | 2 | 2 | 0 | 0 | 0 | 0 | 0 | 0 | 0 | 0 | 0 | 0 |
| **110** | ACTA RADIOL | 1 | 4 | 1 | 4 | 4 | 4 | 4 | 4 | 4 | 4 | 4 | 4 | 4 | 4 | 4 | 0 | 0 | 0 | 0 | 0 | 0 | 0 | 0 | 0 | 0 |
| **111** | J COMPUT ASSIST TOMO | 0 | 0 | 0 | 0 | 0 | 0 | 0 | 0 | 0 | 0 | 0 | 0 | 0 | 0 | 0 | 0 | 0 | 0 | 0 | 0 | 0 | 0 | 0 | 0 | 0 |
| **112** | ULTRASOUND Q | 0 | 0 | 0 | 0 | 0 | 0 | 0 | 0 | 0 | 0 | 0 | 0 | 0 | 0 | 0 | 0 | 0 | 0 | 0 | 0 | 0 | 0 | 0 | 0 | 0 |
| **113** | MED DOSIM | 0 | 0 | 0 | 0 | 0 | 0 | 0 | 0 | 0 | 0 | 0 | 0 | 0 | 0 | 0 | 0 | 0 | 0 | 0 | 0 | 0 | 0 | 0 | 0 | 0 |
| **114** | RADIAT PROT DOSIM | 0 | 0 | 0 | 0 | 0 | 0 | 0 | 0 | 0 | 0 | 0 | 0 | 0 | 0 | 0 | 0 | 0 | 0 | 0 | 0 | 0 | 0 | 0 | 0 | 0 |
| **115** | J CLIN ULTRASOUND | 0 | 0 | 0 | 0 | 0 | 0 | 0 | 0 | 0 | 0 | 0 | 0 | 0 | 0 | 0 | 0 | 0 | 0 | 0 | 0 | 0 | 0 | 0 | 0 | 0 |
| **116** | CONCEPT MAGN RESON A | 1 | 1 | 1 | 0 | 0 | 1 | 0 | 1 | 1 | 0 | 0 | 1 | 0 | 0 | 0 | 0 | 0 | 0 | 0 | 0 | 0 | 0 | 0 | 0 | 0 |
| **117** | INT J RADIAT RES | 0 | 0 | 0 | 0 | 0 | 0 | 0 | 0 | 0 | 0 | 0 | 0 | 0 | 0 | 0 | 0 | 0 | 0 | 0 | 0 | 0 | 0 | 0 | 0 | 0 |

Note: 1 = Active strong, 2 = Active weak, 3 = Passive moderate, 4 = Passive weak, 0 = None.
